# Advancing APT detection through transformer-driven feature learning and synthetic data generation

**DOI:** 10.1038/s41598-026-41317-5

**Published:** 2026-03-02

**Authors:** Le Tran Kim Danh, Cho Do Xuan, Nhan Nguyen Van

**Affiliations:** 1 Information Technology Center, Thuongmai University, Hanoi, Vietnam; 2 Faculty of Information Security, Posts and Telecommunications Institute of Technology, Hanoi, Vietnam; 3 Faculty of Information Technology, Dai Nam University, Hanoi, Vietnam

**Keywords:** Advanced Persistent Threat (APT) detection, Transformer-based feature learning, Conditional Generative Model for Synthesis (CGMS), Synthetic data generation, Data augmentation, Class imbalance, Engineering, Mathematics and computing

## Abstract

Advanced Persistent Threat (APT) detection based on artificial intelligence (AI) platforms has emerged as a dominant trend, has attracted increasing attention in cybersecurity. Nevertheless, two major challenges remain: (i) how to effectively extract discriminative features from complex network traffic flows, and (ii) how to address severe class imbalance caused by the rarity of APT attacks. To tackle these challenges, we propose an integrated pipeline/framework named ET-SDG. The ET-SDG model integrates Transformer-based Feature Learning with a Conditional Generative Model for Synthesis (CGMS). Specifically, the Transformer-based feature learning component combines the ExtraTrees algorithm with a Transformer architecture to select, aggregate, and encode informative flow-level features. To mitigate data imbalance, ET-SDG incorporates CGMS, a cGAN-based synthetic data generation module designed for data augmentation of minority APT traffic. By conditioning the generation process on class labels, CGMS synthesizes representative minority-class samples, aiming to improve the robustness and generalization of the downstream detection model under class imbalance. Across the evaluated benchmarks, ET-SDG shows competitive results and provides modest improvements (approximately 1–4% points, depending on the dataset and metric) relative to the compared baselines.

## Introduction

### Background and motivation

According to^1–4^, Advanced Persistent Threats (APTs) represent one of the most critical and complex challenges in modern cybersecurity. Characterized by their persistence, stealth, and sophistication, APTs are capable of bypassing conventional security mechanisms and remaining undetected for extended periods. Unlike common cyberattacks, these campaigns are often conducted by highly organized threat groups with specific strategic objectives, typically targeting sensitive systems such as governmental networks, financial institutions, or critical infrastructure. Their goals go beyond mere data exfiltration, encompassing operational disruption and even long-term manipulation of internal systems. Through the use of advanced techniques—such as zero-day exploits, lateral movement, and encrypted command-and-control (C2) communications—attackers can sustain unauthorized access and control within the target environment. Consequently, traditional Intrusion Detection Systems (IDS), which mainly rely on rule- or signature-based methods, often fail to identify APT activities effectively.

To address the task of detecting and providing early warnings of APT attacks, several prominent methods using machine learning (ML) and deep learning (DL) for network traffic analysis have been proposed^5–10^. Recently, APT detection models based on flow analysis in network traffic have emerged as an effective approach due to their ability to identify abnormal behaviors with high detail and accuracy. Unlike traditional methods that rely solely on signature-based or rule-based detection, this approach exploits network flow features to detect suspicious behavior patterns, even when the APT uses evasion techniques like unknown malware or advanced attack strategies. By monitoring and analyzing flow characteristics such as connection time, packet frequency, latency, and relationships between IP pairs, this approach can identify anomalies that traditional systems often miss.

However, there are several major challenges in these approaches: Firstly, effectively extracting and representing features from network traffic flows^11–14^. Traditional methods typically rely on handcrafted features such as traffic duration, packet size, or entropy, but these features are not robust enough to reflect the complex relationships between packets within the same network session. Some previous studies have used deep learning models like Recurrent Neural Networks (RNNs) or Long Short-Term Memory (LSTM) networks to extract features from sequential data, but these models suffer from computational inefficiencies, especially when dealing with large-scale network datasets. Secondly, the data imbalance problem^15–21^. Due to the rarity of APT attacks and the sensitive nature of cybersecurity incidents, collecting real-world data is often limited. As a result, most existing datasets are severely imbalanced, with normal network traffic vastly outnumbering attack traffic. When trained on such datasets, models tend to be biased towards the majority class, leading to a high false negative rate during deployment. Methods like SMOTE or random oversampling do not generate truly new data but merely expand existing samples, preventing the model from learning the complex variations of APT attacks.

### Proposed method

To address the two challenges described above—(i) effective feature representation from complex network traffic flows and (ii) severe class imbalance—we develop ET-SDG as an integrated pipeline. ET-SDG follows an ensemble-learning-oriented design and combines feature ranking, contextual encoding, and minority-class augmentation in a unified workflow. In addition, to reduce the risk of overly optimistic estimates, we adopt a leakage-aware evaluation protocol in which data splitting is performed at the IP-pair level, and any feature aggregation and CGMS-based synthesis are applied only within the training partition (or training fold), while the test set remains untouched for evaluation.

Firstly, to efficiently extract and represent informative flow features, ET-SDG differs from several deep learning and graph-based pipelines by combining ExtraTrees with a Transformer-based encoder (ET). ExtraTrees is used to rank and select relevant flow features, while the Transformer encoder models contextual dependencies among the selected features. With its self-attention mechanism, the Transformer can capture both local and global relationships within network traffic, and it supports efficient parallelization and scalability compared with sequential models such as LSTM and BiLSTM.

Secondly, to mitigate the data imbalance issue, ET-SDG incorporates two training-time integration strategies. (1) We introduce CGMS as a synthesis module, which leverages a standard conditional GAN (cGAN) formulation to generate additional minority-class feature vectors for training augmentation. This design aims to improve learning under imbalanced conditions without claiming a new adversarial objective. (2) We employ an attention-based aggregation/classification component to emphasize informative patterns in the learned representations, rather than relying on simple averaging used in some prior methods, thereby improving the expressiveness of the final IP-pair representation for detection.

### Scientific contributions of the paper

Based on the analysis and evaluation presented above, it is evident that the ET-SDG model performs APT attack detection through three main phases: First, the flow features are standardized, extracted, and represented through the ET model. Subsequently, these feature vectors, after encoding, pass through the CGMS model to generate any missing labels. Finally, they are processed through the Attention network to select and highlight the important information before being input into the classification model. Therefore, the key scientific contributions of our paper are as follows:

- First, we develop ET-SDG as an integrated pipeline that combines feature ranking, contextual representation learning, and minority-class augmentation for APT detection. The contribution lies in the validated module integration and the leakage-aware IP-pair–level evaluation protocol, rather than introducing a new learning paradigm.
- Second, we design the ET module (ExtraTrees + Transformer encoder) to enhance flow-level feature representation for APT detection. Experimental results indicate that the ET module achieves competitive performance relative to the considered hybrid baselines on the evaluated metrics.
- Third, we integrate CGMS-based (cGAN) augmentation with an attention-based classification/aggregation component to improve learning under severe class imbalance. The experimental results suggest that this design can improve IP-level APT detection performance compared with the considered alternatives under the evaluated settings.

## Related work

In paper^22^, Cho and colleagues proposed a Multilayer Perceptron (MLP) model for detecting APT attacks based on network traffic analysis. The study constructed an MLP model to learn and analyze anomalies in network traffic in order to identify abnormal activities associated with attacks. The paper reported promising results with an accuracy of up to 92%, significantly outperforming traditional machine learning models. However, the method also has certain limitations. For complex data such as frame-based sequential data like IP traffic, MLP becomes increasingly complicated and expensive to train due to the need for more layers. Moreover, the model cannot capture sequential relationships, which are characteristic of sequential datasets in general and traffic-based detection methods in particular.

The study by Cuong and colleagues^23^ proposed the MCG model—an APT attack detection method based on deep learning combined with graph representation. The model integrates three main components: MLP to extract behavioral features from network flows, CR (Correlated Recursion) to group flows by IP address and construct a relationship graph between IPs, and GAT (Graph Attention Network) to capture contextual relationships between IPs through the Attention mechanism on the graph. The graph construction process allows the model to learn interactions among network entities, thereby representing IPs as vectors in the feature space. Each IP vector is then passed through a classifier to determine whether it is likely to be APT or benign. MCG demonstrated superior performance compared to traditional deep learning models, achieving an overall accuracy of up to 95%. Despite its strong effectiveness in APT detection, the MCG model still has certain limitations. First, the model requires input data to have clearly defined relationships between network entities, especially between IPs, in order to build the relational graph. In cases where connection information is incomplete or network traffic is encrypted, graph construction becomes difficult and may result in the loss of important structural information, thereby reducing model performance. Second, similar to many other graph-based models, MCG does not fully exploit the temporal continuity of APT attacks, which often unfold in multi-stage processes. The lack of global temporal information makes it difficult for the model to detect complex and long-lasting attack sequences.

In paper^24^, the authors proposed the ACG-BT model to enhance the effectiveness of APT attack detection by integrating modern deep learning techniques. The model consists of four main components: Adaptive Contextual (AC), Selective Adversarial Generation (SAG), BiLSTM, and Transformer. Among them, AC plays the role of flexibly adapting to different types of network traffic data, filtering important information while preserving context, thereby improving the ability to detect abnormal behavior patterns. SAG is used to generate simulated attack samples to balance the training dataset and enhance the model’s learning capability when dealing with imbalanced data. The BiLSTM component is responsible for capturing temporal relationships among network events, while the Transformer uses the attention mechanism to learn long-term dependencies and improve parallel processing capabilities. The combination of BiLSTM and Transformer enables the model to both leverage sequential information and enhance the representation of complex contextual patterns in network data. Experimental results show that ACG-BT achieves outstanding performance with an accuracy of 99.45%, recall of 97.85%, and F1-score of 98.35%, improving by 2% to 4% over previous methods across all evaluation metrics.

In^25^, Lu et al. proposed a model named XAPT for APT detection, which integrates three main components. First, a PCA module is used for feature compression and dimensionality reduction. Second, a Bayesian Network is employed to infer and aggregate APT-related behaviors. Third, SHAP-based analysis is applied to assess the contribution and importance of behaviors/features. XAPT was evaluated on two public datasets, namely DAPT2020 and Meta-Alerts, and the reported results indicate promising effectiveness. However, from our perspective, these datasets primarily represent more general cyberattack patterns and alert-level signals rather than realistic APT traffic in operational IDS settings, which may limit the transferability of the reported conclusions to IDS-based APT detection. In^26^, a machine-learning approach combining FastText and SVM was proposed to summarize, extract, and classify APT attacks from Linux payload execution processes. The study follows an APT lifecycle-oriented perspective in Linux environments and evaluates the proposed method against several alternative approaches on a specific dataset. The experimental results suggest that the FastText–SVM pipeline can outperform conventional and hybrid machine-learning baselines in the considered setting.

In^27^, the authors proposed TSE-APT to mitigate two common issues in APT detection—false alarms and limited accuracy—by combining three components: Random Forest (RF), MLP, and BiLSTM. The evaluation was conducted on approximately 5 million records from the CIC-IDS2018 dataset. While the reported classification performance is strong, this evaluation setting may not fully reflect APT characteristics, and it does not explicitly emphasize the complexity and severe imbalance typically observed in CIC-IDS2018. As a result, the applicability of TSE-APT to IDS-based APT detection in realistic deployments remains to be further validated. Ren^28^ investigated APT detection by modeling complex and dynamic network relationships and latent attack patterns during the attack process, proposing a hybrid approach that combines a bidirectional dynamic graph attention mechanism with an improved KAN-based network. The proposed model was evaluated using datasets derived from Common Vulnerabilities and Exposures (CVE) and the National Vulnerability Database (NVD).

Liu et al.^29^ proposed an improved GraphSAGE approach to capture contextual relationships among system entities for early APT detection and alerting in organizations. The authors constructed a provenance graph to handle large-scale data and employed a One-Class SVM to detect anomalous growth with low latency. Experiments on DARPA and StreamSpot datasets indicate improved detection effectiveness and computational efficiency. Sidahmed et al.^30^ addressed three major challenges in APT detection with traditional machine-learning pipelines, including class imbalance, high-dimensional feature spaces, and the scarcity of attack traces in realistic environments, by proposing a multi-component framework integrating transfer learning, explainable AI, contrastive learning, and Siamese networks. The framework was evaluated under multiple scenarios on the DARPA Transparent Computing dataset for detecting APT traces.

## Our methodology for detecting APT attacks

### The workflow of the approach

**Fig. 1:**
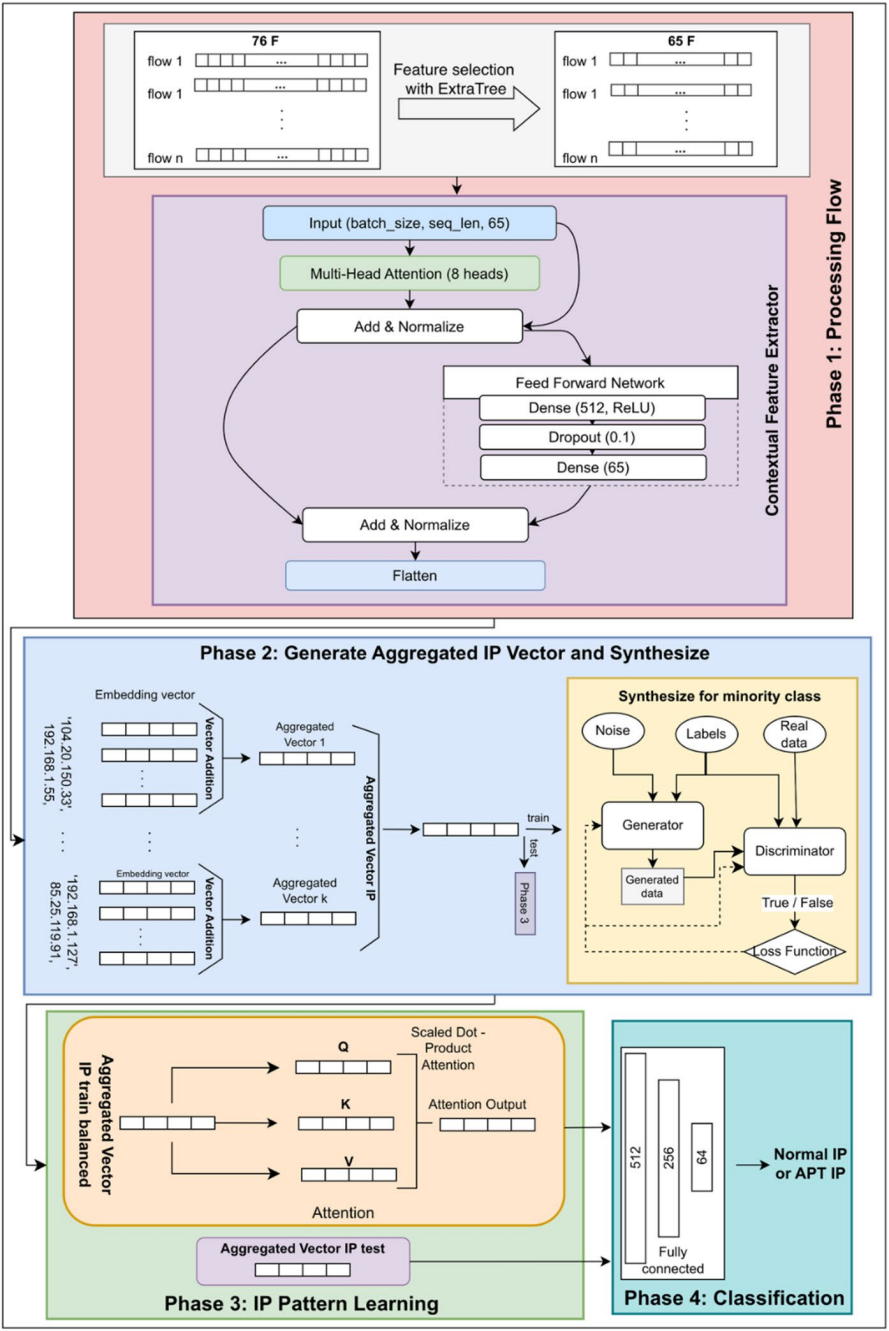
ET-SDG.

Figure 1 below illustrates the working principle of model for APT attack detection.

From Fig. 1, it can be seen that the process in the ET-SDG includes the following phases:

Phase 1: Processing Flow This foundational phase focuses on feature extraction and optimization from network traffic data through two key stages:

- Feature Selection Stage: This initial stage identifies and extracts the most relevant features from network flows. The system begins with 76 features extracted from network traffic data, which are then processed through the ExtraTree algorithm. This algorithm was chosen for its superior performance in handling high-dimensional data and its ability to reduce noise while maintaining feature importance. The process results in a refined set of 65 key features that capture the most significant aspects of network behavior.
- Contextual Feature Extractor Stage: This stage employs a sophisticated Transformer-based architecture to encode and represent the selected features. The process begins with an input layer configured with specific parameters (batch_size, seq_len, 65) to accommodate the selected features. The architecture incorporates an 8-head Multi-Head Attention mechanism, chosen to capture complex relationships across different feature subspaces while maintaining computational efficiency. The Feed-Forward Network consists of two Dense layers (512 and 65 nodes) with ReLU activation, strategically designed to capture hierarchical patterns in the data. A dropout rate of 0.1 helps prevent overfitting while maintaining model robustness. Add & Normalize layers are implemented throughout the network to ensure gradient stability and enhance convergence. The output is a contextualized 65-dimensional feature representation optimized for subsequent processing.

Phase 2: Generate Aggregated IP Vector and Synthesize: This phase consolidates network flow information and addresses data imbalance through two critical stages:

- Aggregated IP Vector Generation: The system aggregates feature vectors from Phase 1 based on source and destination IP address pairs. A median-based aggregation strategy is employed to ensure robustness against outliers while capturing the central tendencies of network behavior. This process generates representative vectors that summarize the behavioral patterns of each IP pair, reducing noise and improving feature quality. After the dataset is split into training and testing sets, aggregated IP vectors are generated independently for each partition. Only aggregated vectors derived from the training set are used in the subsequent synthesis process, while the test set remains untouched for evaluation.
- Minority Class Synthesis: To address the class imbalance issue, this stage utilizes a Conditional Generative Model for Synthesis (CGMS) to generate high-quality feature vectors for minority classes. The generator synthesizes representative samples, while the discriminator ensures statistical consistency with real data. By augmenting the training data with synthesized samples, the model improves its ability to detect rare but significant APT activities, leading to a more balanced and effective detection framework.

Phase 3: IP Pattern Learning and Feature Attention Mechanism: This phase is responsible for learning and recognizing IP behavior patterns by processing aggregated IP vectors through a sophisticated attention mechanism. The system employs Scaled Dot-Product Attention, which dynamically computes the importance of different network features using Query (Q), Key (K), and Value (V) matrices. By applying scaled dot-product operations, the mechanism ensures stable gradient flow during training while allowing the model to focus on the most relevant patterns in network traffic. The computed attention scores guide the model in identifying intricate dependencies across network behaviors, thereby enhancing its ability to detect anomalous activities linked to APT attacks. Since the aggregated IP vectors were split in Phase 2, only the training portion (including both real and synthesized minority class data) is fed into the attention-based learning process. The test data, which remains unaltered, will be used in the final evaluation to assess the generalization capability of the trained model. This structured approach ensures that the model learns effectively while preventing data leakage and preserving realistic classification performance.

Phase 4: Deep Learning-Based Network Traffic Classification: In this phase, the model classifies network traffic patterns by processing the attention-weighted features through a fully connected deep learning architecture. The network consists of multiple layers (512-256-64 nodes) that progressively refine feature representations before making a final classification decision between Normal IP traffic and APT-related IP traffic. Each layer contributes to capturing hierarchical relationships in the data while ensuring computational efficiency. The structured reduction in layer dimensions prevents overfitting by focusing on the most relevant feature attributes. The final classification output is generated using a softmax activation function, assigning probabilities to different traffic categories to ensure precise and reliable detection of APT threats.

The integrated system demonstrates robust performance in distinguishing between normal and APT network traffic, with each phase contributing to the overall accuracy and reliability of the detection process. The architecture’s modular design allows for individual component optimization while maintaining end-to-end performance.

### Processing flow

#### Data preprocessing

The data preprocessing section of the proposed intrusion detection model starts with the investigation and visualization of dataset features. The categorical features of the CIC-flow dataset are converted using label encoding. In the CIC-flow dataset, there are 76 features are float type, and only the label (target feature) is categorical and label encoded. After label encoding, data in each feature of the dataset are normalized using Standard Scaler before passing the datasets to the Extra Tree classifier to calculate the importance of each feature in the dataset. To prevent any form of data leakage, train–test splitting is performed prior to feature aggregation. Feature aggregation is then conducted independently on the training and test partitions, ensuring that aggregated IP-level representations in the training set are computed solely from training flows, while aggregated representations in the test set are computed solely from test flows. Consequently, no information from the test set contributes to feature construction or model training.

#### Feature selection based on ExtraTrees

In calculating feature importance, impurity-based variables can be evaluated using tree-based cost estimators, allowing for the removal of irrelevant features. We adopted the innovative ensemble feature evaluation method proposed by^31^, known as the ExtraTree classifier, which is capable of identifying feature importance. This method led us to select 65 significant features from the CIC-flow dataset, while discarding the remaining unnecessary features. The Extra Tree classifier uses an ensemble of randomized decision trees to assess the importance of each feature based on the Gini Index, producing de-correlated trees through random feature selection^2,32^. Many de-correlated decision trees are produced by this random feature selection. The numerical criteria (Gini Index if the Gini Index is utilized in the forest design) that were implemented to determine which features ought to be split are scaled to give every feature an aggregate decline. The outcomes of the test are known as the Gini importance of features. After grading all features in terms of their Gini Importance in the lowest to the highest order, the user decides on the top number of features. The determining element in this situation is going to be Information Gain. Start by calculating the Entropy of the data.

1 $$\:Entropy\left(S\right)={\sum\:}_{i=1}c-pi\mathrm{log}2\left(pi\right)$$

c - Represents the total number of unique target labels in the target variable, $\:p$i - Represents the Proportion of rows in a data frame, i- output label Gain at the initial tree in the forest is calculated using:

2 $$\:G\mathrm{a}\mathrm{i}\mathrm{n}\:\left(\mathrm{S},\mathrm{A}\right)=\:\mathrm{E}\mathrm{n}\mathrm{t}\mathrm{r}\mathrm{o}\mathrm{p}\mathrm{y}\:\left(\mathrm{S}\right)-\:\sum\:\mathrm{v}{\upepsilon\:}\mathrm{V}\mathrm{a}\mathrm{l}\mathrm{u}\mathrm{e}\mathrm{s}\left(\mathrm{A}\right)\:\left|\mathrm{S}\mathrm{v}\right|\:/\:\left|\mathrm{S}\right|\:\mathrm{E}\mathrm{n}\mathrm{t}\mathrm{r}\mathrm{o}\mathrm{p}\mathrm{y}\:\left(\mathrm{S}\mathrm{v}\right)$$

A - Individual feature in the data frame.

Every decision tree computes the gain of every feature within the data frame. For example:

- Gain (G1) = (Entropy (S), A1) represents the gain of the feature named A1 at Decision tree D1.
- Gain (G2) = (Entropy (S), A2) represents the gain of the feature named A2 at Decision tree D2.
- Gain (Gn) = (Enteropy (S), An) represents the gain of the feature named An at the Decision tree Dn.

When this process is completed, the total Gain value of each feature is calculated, and the features with the highest Gain values are considered important.

#### Contextual feature extractor using transformer

The contextual feature extractor consists of three main layers: Attention Layer, Residual-Normalization Layer, and Feed-Forward Layer^33^. Each layer performs specific transformations, enhancing the input representation through contextual modeling, stabilization, and feature enrichment. This section provides a modular breakdown, supported by pseudo-code and hierarchical visualization.

##### Attention layer

The Attention Layer models global dependencies among features by computing relationships between all pairs of inputs using Multi-Head Attention (MHA).

- Mathematical Definition:
- Here, $\:{d}_{k}$denotes the dimensionality of the key/query vectors (per head). The term $\:\sqrt{{d}_{k}}$is the scaling factor in scaled dot-product attention. Each attention head computes a scaled dot-product attention:

3 $${\mathrm{Attention}}(Q,K,V) = {\mathrm{softmax}}\left( {\frac{{QK^{{\mathrm{T}}} }}{{\sqrt {d_{k} } }}} \right)V$$

Where $Q_{i} ,K_{i} ,V_{i}$ are linear projections of *X* for head *i*-th:

4 $$Q_{i} = XW_{i}^{Q} ,\quad K_{i} = XW_{i}^{K} ,\quad V_{i} = XW_{i}^{V}$$

The outputs of *h* attention heads are concatenated and projected:

5 $${\mathrm{MHA}}(X) = {\mathrm{Concat}}({\mathrm{head}}_{1} , \ldots ,{\mathrm{head}}_{h} )W^{O}$$

With: $${\mathrm{head}}_{i} = {\mathrm{Attention}}(Q_{i} ,K_{i} ,V_{i} )$$

Insights:

- The softmax function produces attention weights that resemble a Bayesian posterior, determining each token’s importance in context.
- The attention mechanism captures global relationships, effectively modeling dependencies across the entire sequence.

The pseudocode below illustrates the operating principle of this network.

**Figure:**
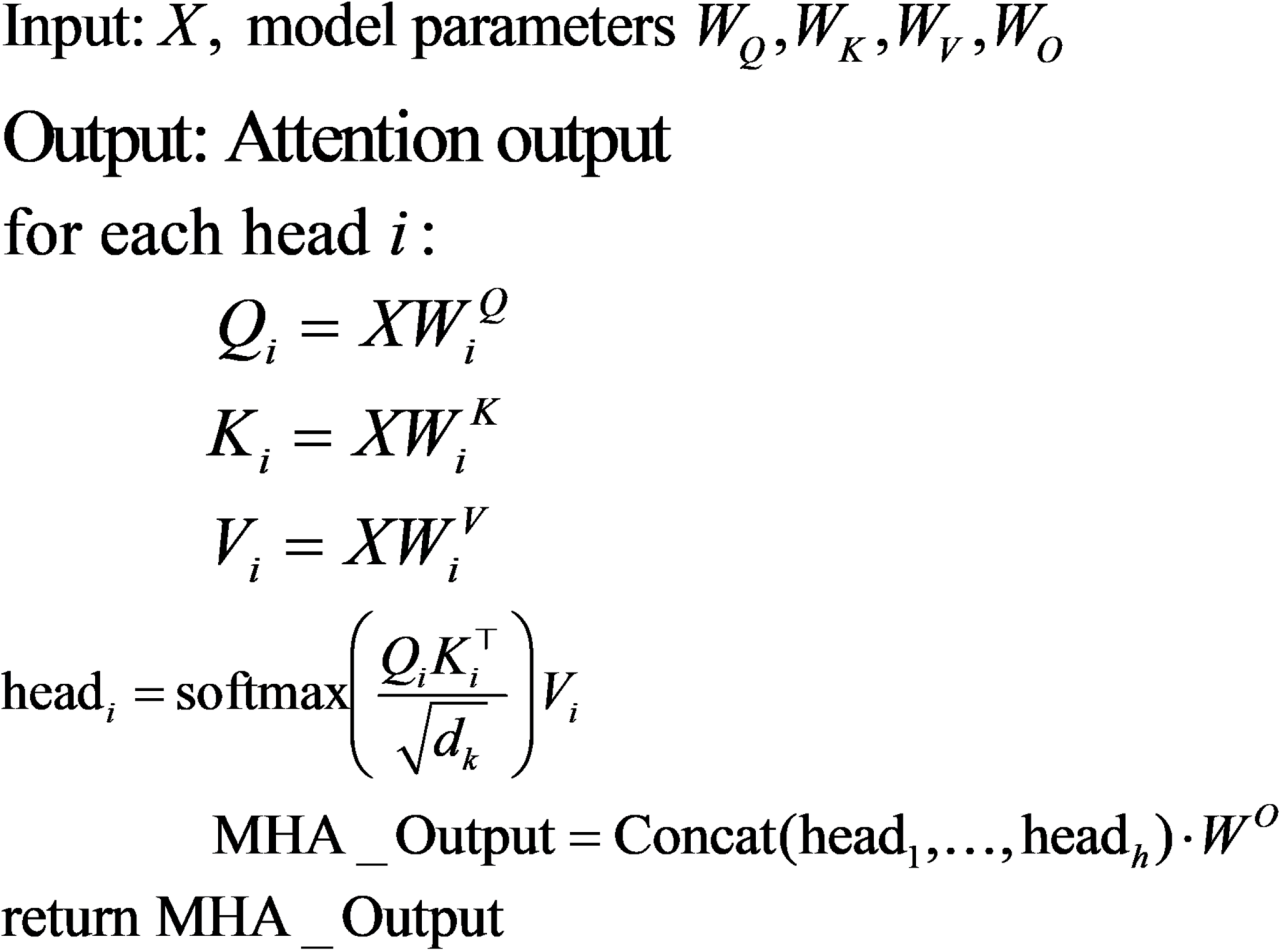

##### Residual-normalization layer

The Residual-Normalization Layer stabilizes the learning process and preserves low-level input signals through residual connections and layer normalization.

1. Residual Connection:
  6 $${\mathrm{Residual}}(x,f) = x + f(x)$$
  Adds the input *x* to the output of the sub-layer $f(x)$, ensuring gradient flow and preventing vanishing gradients.
2. Layer Normalization:
  7 $${\mathrm{Norm}}(x) = \frac{{x - \mu }}{{\sqrt {\sigma ^{2} + \varepsilon } }}$$
  Where *μ* and $\sigma ^{2}$ are the mean and variance of *x*.

Insights:

1. Statistical View: Layer normalization reduces internal covariate shift by standardizing inputs.
2. Geometric View: Residual connections maintain the geometric properties of the input, while LayerNorm projects features onto a hypersphere.

To gain a better understanding of the algorithm, observe the following pseudocode.

**Figure:**
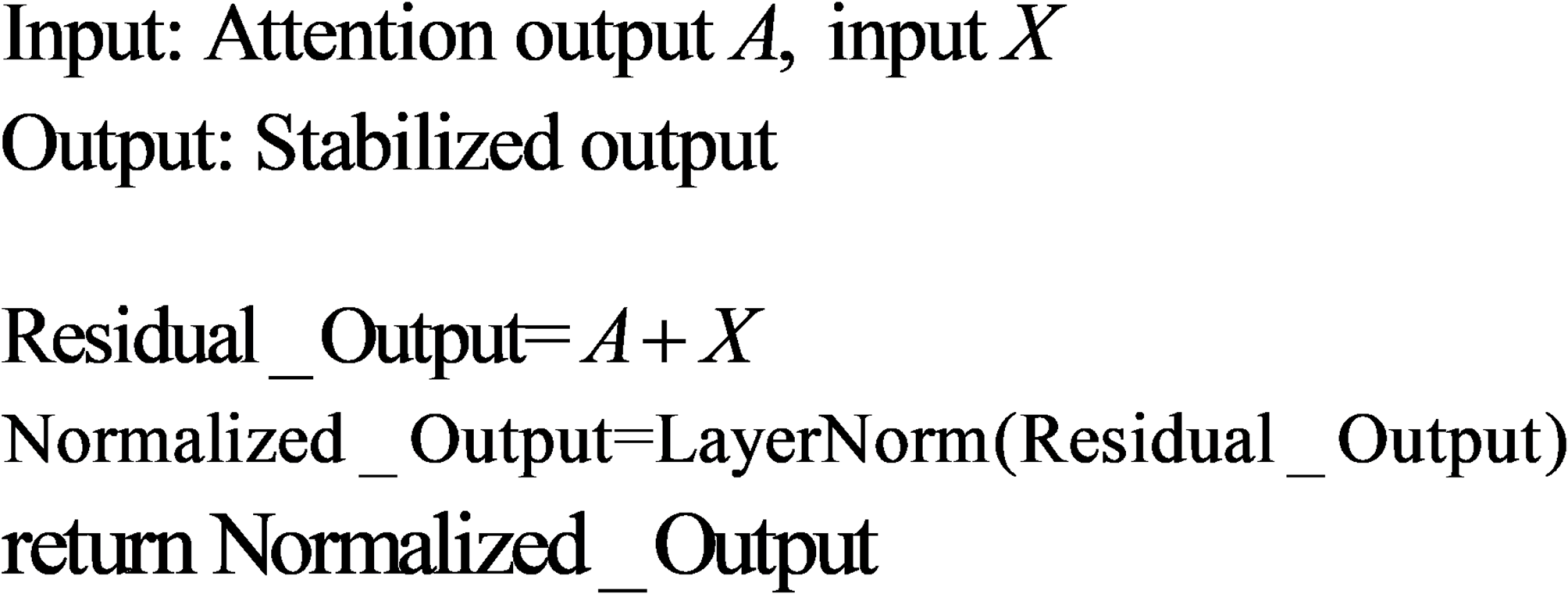

##### Feed-forward layer

The Feed-Forward Layer applies two dense layers with a non-linear activation function to enrich feature representations.

1. Transformation: Projects the input to a higher-dimensional space:
  8 $$x^{\prime} = {\mathrm{ReLU}}(xW_{1} + b_{1} )$$
2. Projection Back: Reduces the representation back to the original dimensionality:
  9 $${\mathrm{FFN}}(x) = x^{\prime}W_{2} + b_{2}$$

Insights:

1. The FFN enhances the model’s capacity by capturing non-linear feature interactions.
2. It acts as a kernel-like approximation, projecting features into richer spaces.

The following pseudocode details the operation of this model.

**Figure:**
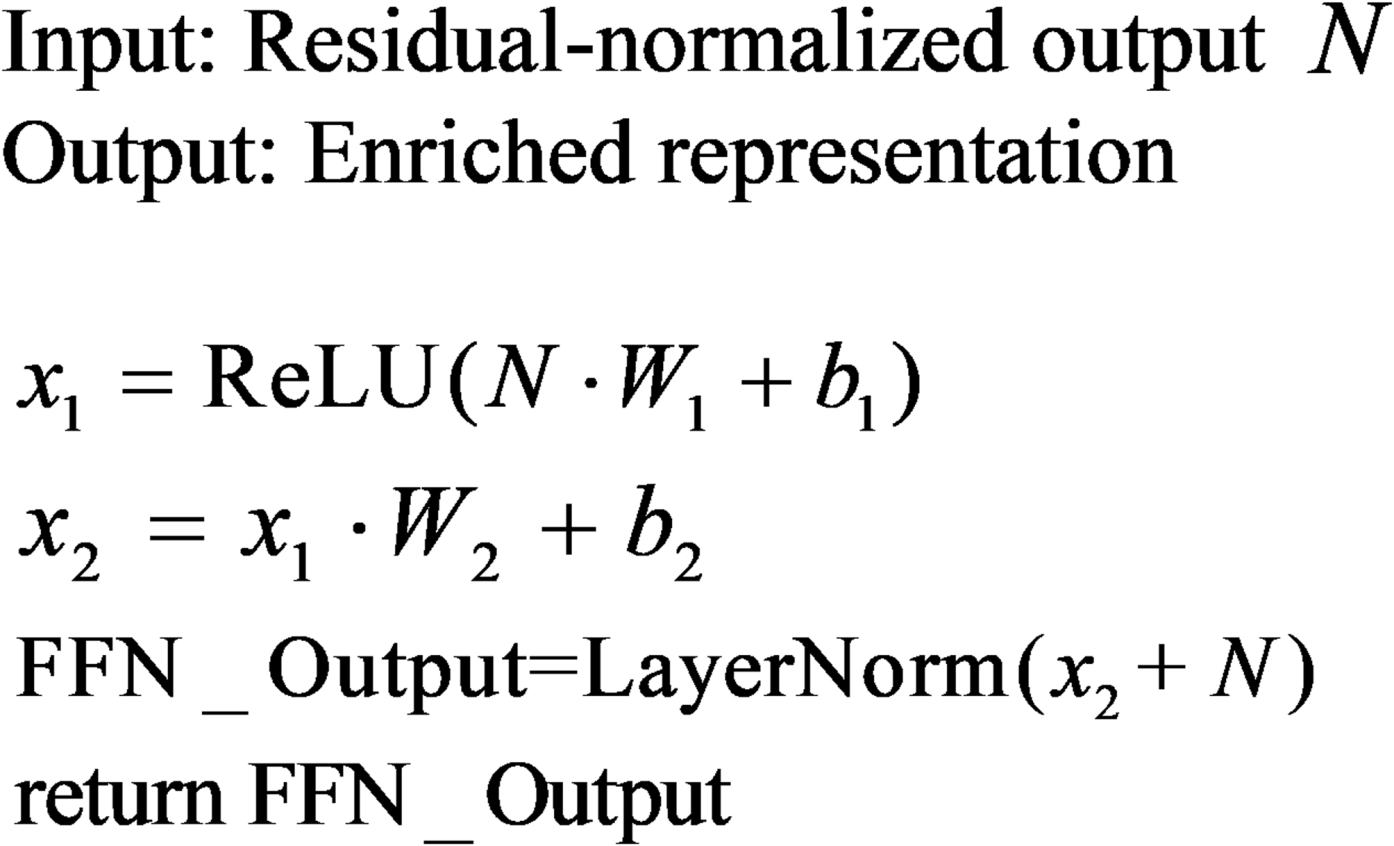

##### Hierarchical modular flow

The flow of the contextual feature extractor is summarized as follows:

1. Attention Layer: Captures global dependencies via multi-head attention:
  10 $$A = MHA(X)$$
2. Residual Connection and Normalization (Post-Attention):
  11 $$R_{1} = {\mathrm{Norm}}(X + A)$$
3. Feed-Forward Layer:
  12 $$F = {\mathrm{FFN}}(R_{1} )$$
4. Residual Connection and Normalization (Post-FFN):
  13 $$Z = {\mathrm{Norm}}(R_{1} + F)$$

The hierarchical diagram is as follows:

**Figure:**
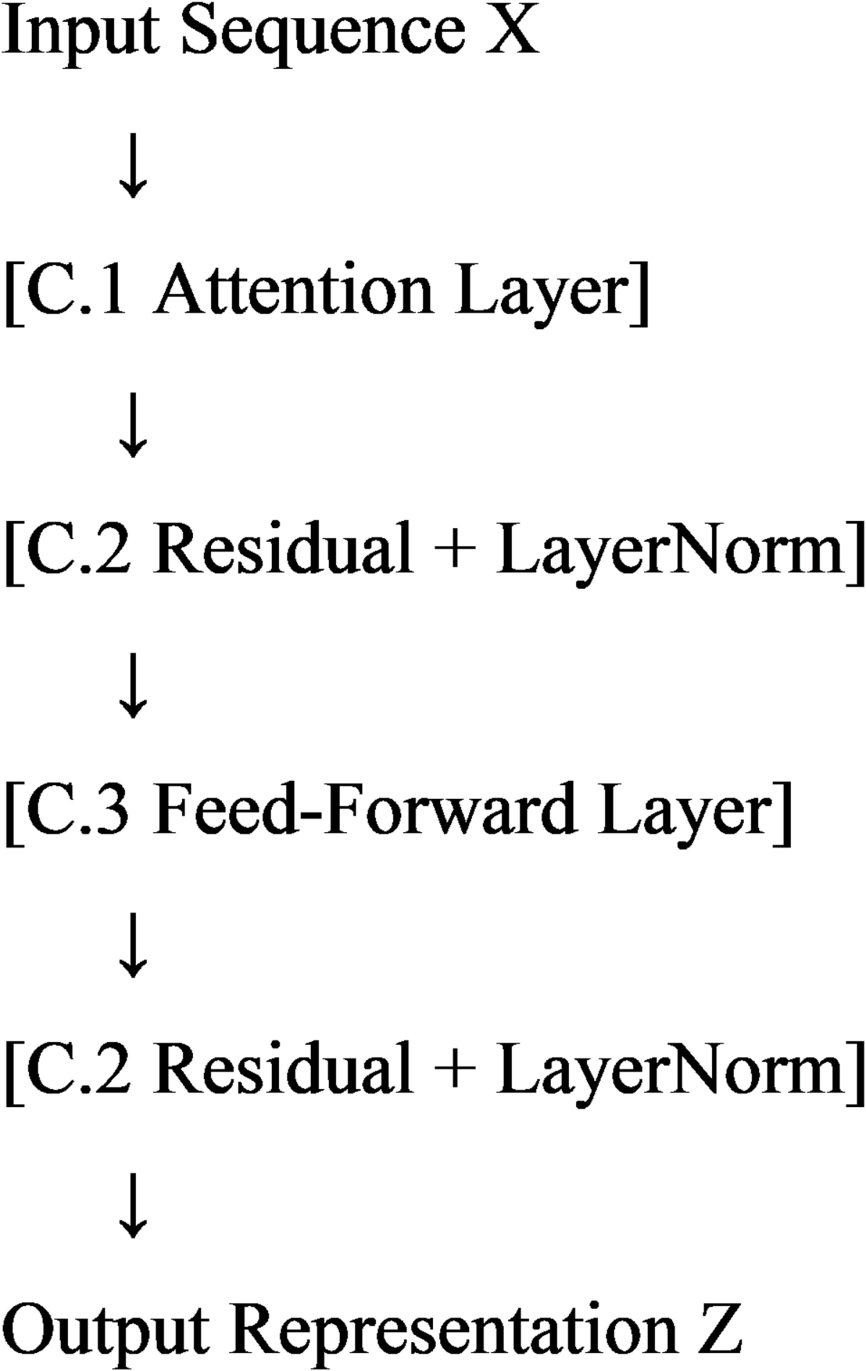

### Generate aggregated IP vector and synthesize

#### Generate aggregated IP vector

The process of generating an IP Frame and extracting IP features can be summarized as follows: After the embedding process based on the Transformer model is completed, the embedding vectors with the same source IP and destination IP are aggregated together to form IP frames. The result of this aggregation is a single vector, which includes the most prominent features of the frame block after combining. This context vector represents the important characteristics corresponding to each original frame.

#### Synthesize for minority data

##### Introduction to conditional generative model for synthesis

In this phase, we introduce the Conditional Generative Model for Synthesis (CGMS) as a cGAN-based synthetic data generation module within the ET-SDG pipeline, designed specifically for data augmentation of minority APT traffic.

CGMS adopts a standard conditional GAN (cGAN) formulation, in which class label information is incorporated as a conditioning variable in both the generator and the discriminator, following the conditional generation paradigm introduced in^34^. By conditioning the generation process on label information, CGMS focuses on synthesizing minority-class APT traffic representations, thereby alleviating class imbalance and improving the effectiveness of downstream detection.

This design enables controlled synthesis of minority-class samples without relying on explicit manual resampling heuristics. Specifically, both the Generator and the Discriminator receive an additional conditioning input Y, representing the target class to be synthesized. As a result, the training process is guided toward generating samples that are consistent with the desired class label while preserving the underlying data distribution. The two core components of CGMS can be summarized as follows.

14 $$G:\mathbb{Z} \times {\mathcal{Y}} \to {\mathcal{X}},\quad D:{\mathcal{X}} \times {\mathcal{Y}} \to [0,1]$$

Where: *G* (Generator module) takes as input a noise vector $z \in \mathbb{Z}$ and label $y \in {\mathcal{Y}}$, and then generates data $\hat{x} \in {\mathcal{X}}$, *D* (Discriminator module) takes as input data (which could be real or fake) along with label *Y* and outputs a prediction value in the range [0, 1].

The CGMS module is trained exclusively on minority-class samples from the training partition after data splitting and aggregation. Synthetic samples generated by CGMS are added only to the training set for subsequent classifier learning. At no stage does CGMS access test data or aggregated representations derived from the test set, ensuring strict separation between training and evaluation data.

##### Objective function

Based on the theoretical foundation of generative adversarial models, the objective function is adjusted to reflect the conditional nature as follows:

15 $$\min _{G} \max _{D} V(D,G) = {\mathbb{E}}_{{x,y\sim p_{{{\mathrm{data}}}} (x,y)}} \left[ {\log D(x,y)} \right] + {\mathbb{E}}_{{z\sim p_{z} (z),{\kern 1pt} y\sim p_{y} (y)}} \left[ {\log \left( {1 - D\left( {G(z,y),y} \right)} \right)} \right]$$

Where: $(x,y)$ is the data distribution sampled from the real data $\:{P}_{data}(x,y)$, $z \in \mathbb{Z}$ is the noise distribution (Gaussian), $\:y$ is the label ${\mathcal{Y}}$ distribution from the training data.

When implementing training, the objective function is separated into two distinct loss functions:

- Loss for the Discriminator (D):
  16 $$J_{D} = - \frac{1}{{2m}}\left( {\sum\limits_{{i = 1}}^{m} {\log } D(x_{i} ,y_{i} ) + \sum\limits_{{i = 1}}^{m} {\log } \left( {1 - D\left( {G(z_{i} ,y_{i} ),y_{i} } \right)} \right)} \right)$$

- Loss for the Generator (G):
  17 $$J_{G} = - \frac{1}{m}\sum\limits_{{i = 1}}^{m} {\log } D\left( {G(z_{i} ,y_{i} ),y_{i} } \right)$$

Where *m* is the batch size, $\:\left({x}_{i},{y}_{i}\right)$ are randomly extracted from the real data distribution, and $\:\left({z}_{i}\right)$ is sampled from a noise distribution (typically Gaussian or Uniform). We emphasize that CGMS does not introduce a new GAN objective or adversarial training paradigm. Instead, CGMS denotes a system-level synthesis component that leverages the standard cGAN objective and integrates it into our IP-level aggregation and downstream detection pipeline.

##### Training process

To apply this method in our paper, the research team followed the structured process outlined in Algorithm 1. Specifically, Algorithm 1 details each step of initialization, training, and synthetic data generation, while simultaneously optimizing both the generator and discriminator in an iterative manner. As a result, the generator gradually improves its ability to generate data samples, while the discriminator enhances its efficiency in distinguishing real data from generated data.

**Algorithm 1:**
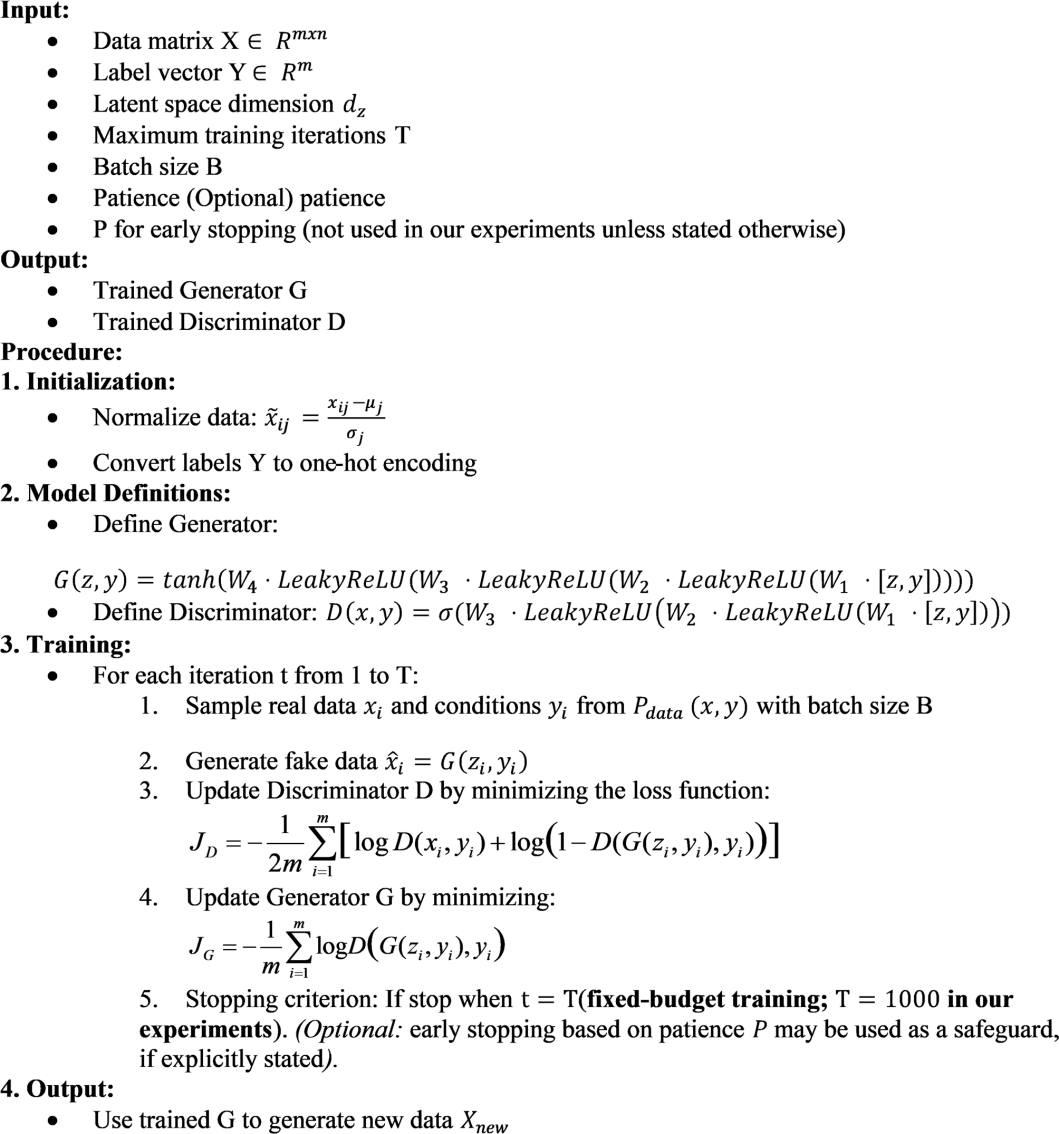
Conditional generative model for synthesis using a cGAN core.

From Algorithm 1, the process begins by normalizing the input data and converting labels into one-hot vectors. The generator and discriminator are then defined, both incorporating label information $\:y\:$to condition the synthesis and discrimination processes. Training proceeds iteratively: at each iteration, a minibatch of real samples is drawn, conditioned fake samples are generated by $\:G$, the discriminator $\:D\:$is updated to distinguish real from generated data, and the generator $\:G\:$is updated to improve the realism of synthesized samples. After training, the optimized generator is used to synthesize additional minority-class samples for training augmentation.

To improve reproducibility, we explicitly specify the training configuration used in our experiments. CGMS (cGAN-based) is trained with batch size $\:B=128\:$under a 1:1 discriminator-to-generator update ratio (one update of $\:D\:$followed by one update of $\:G\:$per iteration), using Adam optimizers with fixed learning rates $\:\left({\eta\:}_{G}=4\times\:{10}^{-4},{\eta\:}_{D}=1\times\:{10}^{-4},{\beta\:}_{1}=0.5,{\beta\:}_{2}=0.999\right)$. Unless otherwise stated, training runs for a fixed budget of $\:T=1000\:$iterations and no learning-rate scheduling is applied.

Early stopping can be incorporated as an optional safeguard; however, in the experiments reported in this paper, CGMS training is terminated upon reaching the fixed iteration budget $\:T=1000$ to ensure a deterministic and easily reproducible protocol.

### IP pattern learning using the attention network

At this phase, feature vectors of frames are passed through the Attention network to find and highlight important features, instead of averaging them as done by other classification methods. Specifically, this process is as follows: First, after the Transformer model processes frame networks, all hidden states of the network are retained by flexibly combining processing blocks including Q, K, V, etc. Thus, the Attention network helps create an output vector where IP features are aggregated and highlighted by processing and evaluating the frame network. Clearly, there is a significant difference between the output data of the BiLSTM model and that of the Attention network. Specifically, in the output vector of BiLSTM, features near the end are extracted and stored more than those at the beginning. In the output vector of the Attention network, important features can appear anywhere because this vector contains features collected based on the entire dataset. From here, it can be seen that with the support of the Attention network, important IP features based on the flow network are extracted and emphasized.

### Classification

At this stage, the focus shifts from feature learning to utilizing them in the classification model to make accurate predictions on the processed data. The deep neural network we used consists of 3 fully connected Dense layers with sizes 256, 64, and 32, respectively. The input to the network consists of attention vectors, representing the most important features of the data after being refined by the Attention mechanism, and the output of the network contains 2 nodes corresponding to the two classes: IP Normal and IP APT.

## Experiments and evaluation of the ET-SDG

### Experimental dataset

The experimental data were collected and analyzed from 29 network traffic files in the Malware Capture CTU-13 dataset, which contains six types of malicious codes from APT attacks, including Andromeda, Cobalt, Cridex, Dridex, Emotet, and Gh0stRAT^38^. The clean network traffic dataset in this paper was extracted from the e-government server of the Quang Nam Province Department of Information and Communications Soc Trang Province^39^, under the scientific research project KC.01.05/16–20 of the Ministry of Science and Technology of Vietnam. This dataset was collected on July 27, 2019.

The detailed quantity of labels in the experimental dataset is as follows:

- 1,671,280 flows, including 799,466 normal flows and 871,814 APT flows.
- 1,884 IP addresses, including 242 pairs of APT attack IPs and 1,642 pairs of normal IPs.

The dataset was divided into training and test sets with a ratio of 8:2 based on the number of IP addresses, where the training set was used to train the model, the validation set (20% of the training set) was used to develop the model during the training phase, and the test set was used to evaluate the model’s effectiveness. All evaluations presented in this paper were conducted on the test set—the dataset the model had never “seen” during the training phase. In addition, we report statistically robust results under 5-fold stratified cross-validation (Sect.  4.5.2), where each fold follows the same 80/20 protocol and the validation set is drawn only from the corresponding training fold.

### Evaluation criteria

The model is evaluated using four primary metrics during the experiments: Accuracy, Precision, Recall, and F1-score. The general formulas for these metrics are as follows.

Accuracy:

18 $$\:Accuracy=\frac{TP+TN}{TP+TN+FP+FN}\times\:100\%$$

Precision:

19 $$\:Precision=\frac{TP}{TP+FP}\times\:100\%$$

Recall:

20 $$\:Recall=\frac{TP}{TP+FN}\times\:100\%$$

F1-score:

21 $$\:F1=\frac{2\times\:Precision\times\:Recall}{Precision+Recall}$$

Where, TP is true positive; FN is false negative; TN is true negative; FP is false positive.

### Experimental setup and implementation details

This section describes the experimental setup and implementation details of the proposed ET-SDG framework to ensure reproducibility of the reported results.

Raw network flow features are first preprocessed using standard normalization with zero mean and unit variance. The dataset is then divided into training and test sets using a stratified 80:20 split at the IP-pair level. To prevent information leakage, feature aggregation is performed after data splitting and is applied independently to the training and test partitions, ensuring that aggregated representations in the training set are computed solely from training flows.

To address class imbalance, the Conditional Generative Model for Synthesis (CGMS) is trained exclusively on minority-class samples from the training set. Synthetic samples generated by CGMS are added only to the training data for augmentation. The Transformer-based feature extraction module and the attention-based classifier are subsequently trained on the augmented training set, while the test set remains untouched and is used solely for evaluation.

All architectural configurations and hyperparameters of the ET-SDG framework, including the Transformer-based feature extractor, CGMS module, and attention-based classifier, are summarized in Table 1. Hyperparameters are selected via grid search conducted on the training data only, with PR-AUC used as the primary selection criterion due to the imbalanced nature of the APT detection task. A single Transformer layer is adopted, as deeper configurations did not provide additional performance improvements in preliminary experiments.

**Table 1:** Configuration and hyperparameters of the proposed ET-SDG framework.

Module	Component	Hyper-parameter	Value/description
Phase 1: Flow Processing	Feature Selection	Initial feature size	76 flow-level features
		Feature selector	ExtraTrees
		Selected features	60 (optimal, tested range: 10–76 step: 5)
	Transformer-based Feature Extractor	Input shape	(batch_size, seq_len, 65)
		Number of attention heads	8 (tested range: 2, 4, 8, 16, 32)
		Number of Transformer layers	1
		Feed-forward dimensions	512 → 65
		Activation function	ReLU
		Dropout rate	0.1
		Normalization	Layer Normalization
		Output dimension	65
Phase 2: Aggregation & CGMS	IP-level Aggregation	Aggregation strategy	Median aggregation by source–destination IP pair
		Aggregation scope	Applied independently to training and test sets
	CGMS (cGAN-based)	Conditioning variable	Class label (Normal / APT)
		Latent dimension	100
		Generator architecture	[100 + class] → 256 → 512 → 1024 → output_dim
		Generator activation	LeakyReLU(0.2), BatchNorm1d, Tanh (output)
		Discriminator architecture	[output_dim + class] → 256 → 128 → 1
		Discriminator activation	LeakyReLU(0.2), Dropout(0.3), Sigmoid
		Generator learning rate	0.0004
		Discriminator learning rate	0.0001
		Optimizer	Adam (β₁ = 0.5, β₂ = 0.999)
		Loss function	Binary Cross-Entropy
		Batch size	128
		Training epochs	1000
		Training scope	Minority-class samples from training set only
Phase 3: IP Pattern Learning	Attention Mechanism	Attention type	Scaled Dot-Product Attention
		Query / Key / Value dimension	40 / 40 / 40
		Attention activation	Softmax
Phase 4: Classification	Fully Connected Network	Hidden layers	512 → 256 → 64
		Activation function	ReLU
		Output layer	1 neuron, Sigmoid
		Optimizer	Adam
		Loss function	Binary Cross-Entropy
		Batch size	32
		Training epochs	100
Training & Evaluation	Data split	Train / test	80% / 20%
		Cross-validation	5-fold StratifiedKFold
		Hyperparameter selection	Grid search on training set
Implementation Environment	Platform	Execution environment	Google Colab Free Tier
		Execution mode	CPU-only
		CPU	Intel Xeon (virtualized), 2 vCPU cores
		System RAM	~ 12.7 GB
		Storage	~ 100 GB temporary session storage
		GPU	Not used
	Software stack	Programming language	Python 3.10
		Deep learning framework	PyTorch 2.0+
		ML & preprocessing	scikit-learn 1.3+, NumPy 1.24+, Pandas 2.0+, Matplotlib 3.7+, Seaborn 0.12+, Joblib 1.3+

To evaluate robustness and statistical stability, a 5-fold stratified cross-validation protocol is employed in selected experiments. All experiments are implemented in Python using PyTorch and scikit-learn and executed on the Google Colab Free Tier in CPU-only mode. The experimental environment consists of a virtualized Intel Xeon CPU with 2 vCPU cores and approximately 12.7 GB of system memory. No GPU acceleration is used in any experiment. This configuration is sufficient to reproduce all reported results and reflects a commonly accessible computing environment.

### Experimental scenarios

To demonstrate the effectiveness of the ET-SDG, we will evaluate the ET-SDG model based on several research questions (RQ) as follows:

- RQ1: How has the ET-SDG model addressed the feature selection problem for flow in network traffic?
- To answer this question (ARQ), we will conduct experiments under the following scenarios:
  - ARQ1.1: Compare the performance of the ET model with several other hybrid models, including CNN-LSTM, CNN-BiLSTM, LSTM-Attentions, and BiLSTM-Attentions.
  - ARQ1.2: Compare the effectiveness of each component in the ET model. This scenario aims to demonstrate why the ET model was chosen instead of other models.
- RQ2: How has the ET-SDG model improved APT IP classification performance?
- To answer this question, we will perform two main scenarios:
  - ARQ2.1: Compare the CGMS data augmentation method with other CGMS-based synthetic data generations, including SMOTE, Variational Autoencoders (VAEs), and traditional GAN models. The goal of this experiment is to evaluate whether the attack samples generated by CGMS contribute to improving classification performance and model generalization in imbalanced data scenarios.
  - ARQ2.2: Evaluate the effectiveness of each component in the combined CGMS-Attention model.
- RQ3: Does the ET-SDG model outperform existing APT detection methods?

To validate the effectiveness of the model, we will compare ET-SDG with current advanced APT detection models. The models compared include methods based on GCN, deep learning anomaly detection models, and hybrid machine learning - deep learning methods.

### Experimental results

The 5-fold cross-validation protocol described in Sect.  4.5.2 is primarily introduced to assess the robustness and statistical stability of the proposed ET-SDG framework and to rule out the effect of a favorable train–test split. For baseline methods, the reported results follow the experimental settings, data splits, and evaluation protocols described in their respective original studies or publicly available implementations. Re-running all baseline models under the same cross-validation protocol would require substantial reimplementation and careful hyperparameter retuning to ensure fair comparison, which is beyond the scope of this study. Importantly, the relative performance trends between ET-SDG and baseline methods are consistent across multiple evaluation metrics, suggesting that the conclusions drawn in this section do not hinge on a particular data partition. Nevertheless, evaluating selected strong baselines under the same cross-validation protocol remains an interesting direction for future work.

#### Evaluation of the ET-SDG model in feature extraction for flow

##### Experimental results for ARQ1.1: evaluating the effectiveness of ET in feature extraction from flow

Table 2 below lists some results comparing the ET model with other research and models in the task of feature extraction from flow data.

**Table 2:** Comparison of performance of different models in feature extraction from network flow data.

Model	Accuracy	Precision	Recall	F1-Score
Cnn-Lstm-SDG	0.9759	0.9562	0.9576	0.9563
Cnn-Bilstm-SDG	0.9655	0.9658	0.9655	0.9657
Lstm-Attentions-SDG	0.9788	0.9788	0.9788	0.9788
Bilstm-Attetions-SDG	0.9682	0.9707	0.9682	0.9690
Our	0.9933	0.9963	0.9890	0.9926

As summarized in Table 2, the ET-based model demonstrates consistently strong performance across all evaluation metrics, with accuracy and F1-score values approaching the upper range of the evaluation scale. These results indicate that the proposed feature extraction strategy is effective in capturing discriminative patterns from network flow data. Among the baseline approaches, the LSTM–Attention–SDG model achieves the most competitive performance, with all evaluation metrics lying in the 0.97xx range. This observation suggests that incorporating attention mechanisms into sequential architectures can enhance the quality of learned flow representations. Nevertheless, its overall performance remains below that of the ET-based configuration.

The CNN–LSTM–SDG model exhibits comparatively weaker results, particularly in terms of F1-score, reflecting limitations in modeling complex and long-range dependencies inherent in network flow data. Similarly, the CNN–BiLSTM–SDG model attains lower performance across most metrics. Although BiLSTM is capable of modeling bidirectional temporal dependencies, it does not fully exploit spatial characteristics of flow-level features. The BiLSTM–Attention–SDG variant provides a modest improvement; however, the attention mechanism alone does not completely resolve the challenges of learning complex spatio-temporal interactions.

Overall, the results reported in Table 2 indicate that the ET-based model provides a consistent performance advantage over the compared methods, with improvements on the order of 10⁻² depending on the evaluation metric. These findings highlight the effectiveness of combining ExtraTree-based feature selection with Transformer-based representation learning for flow-level feature extraction.

**Fig. 2:**
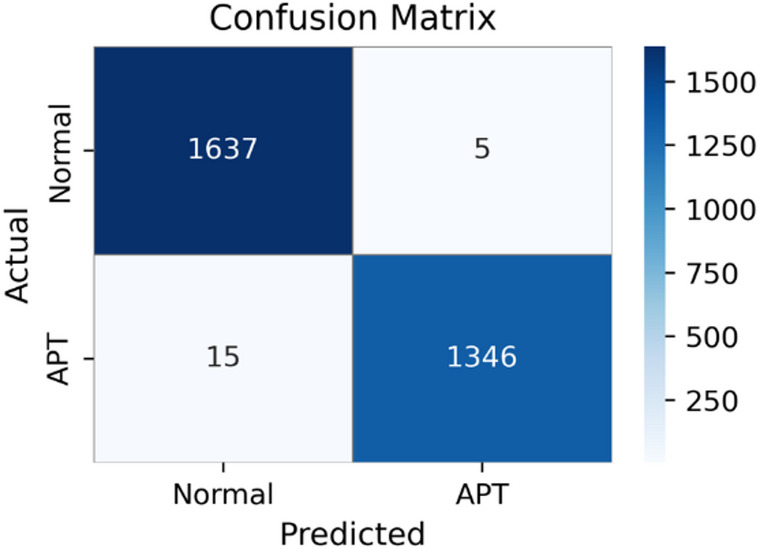
Aggregated confusion matrix of ET-SDG model under 5-fold stratified cross-validation, showing consistently low false positives and false negatives.

Figure 2 presents the aggregated confusion matrix across five folds, showing robust detection performance for APT attacks. Specifically, the model correctly classifies 1,637 Normal samples and 1,346 APT samples, while producing only 5 false positives and 15 false negatives. These results are consistent with the quantitative metrics reported in Table 2, reflecting strong overall classification performance and effective handling of class imbalance.

In particular, minimizing false negatives is critical in APT detection, as undetected attacks may persist in the system for extended periods. The relatively small number of false negatives observed in Fig. 2 suggests that the ET-SDG is capable of reliably identifying malicious activities.

From this experimental scenario, it can be concluded that combining ExtraTree-based feature selection with the Transformer-based learning module enables effective representation learning from flow data, resulting in strong detection capability and improved generalization compared with alternative feature extraction approaches.

##### Experimental results for ARQ1.2: evaluating the effectiveness of each component in the ET model

First: Replacing ExtraTree with other dimensionality reduction models.

In this scenario, we evaluate the effectiveness of replacing the ExtraTree component in the ET model with other dimensionality reduction methods, including AutoEncoder (AE) and Principal Component Analysis (PCA), with all methods using a fixed output dimension of 65. The Transformer component remains unchanged across all experiments. The results are presented in Table 3 below.

**Table 3:** The impact of dimensionality reduction methods on ET model performance.

Dimension: 65	Accuracy	Precision	Recall	F1-Score
AE	0.9735	0.9748	0.9735	0.9739
PCA	0.9788	0.9793	0.9788	0.9790
without ET	0.9761	0.9770	0.9761	0.9764
Our	0.9933	0.9963	0.9890	0.9926

The results in Table 3 indicate that removing ExtraTree (without ET) and using only the Transformer still maintains relatively strong performance, with Accuracy = 0.9761 and F1 = 0.9764. However, these values remain lower than those achieved by the full ET model, suggesting that ExtraTree contributes positively to improving input feature quality. When ExtraTree is replaced with AutoEncoder (AE), performance further decreases, with Accuracy = 0.9735 and F1 = 0.9739. This observation suggests that although AE is effective at compressing data, it focuses primarily on reconstruction rather than task-specific feature selection, which may result in retaining less informative or noisy features. Among the alternative approaches, PCA achieves the strongest performance, with Accuracy = 0.9788 and F1 = 0.9790. By projecting data onto principal components with the largest variance, PCA reduces dimensionality effectively; however, it does not explicitly consider feature importance with respect to the classification objective. Consequently, while PCA outperforms AE and the Transformer-only variant, it still does not match the performance of ExtraTree-based feature selection.

**Fig. 3:**
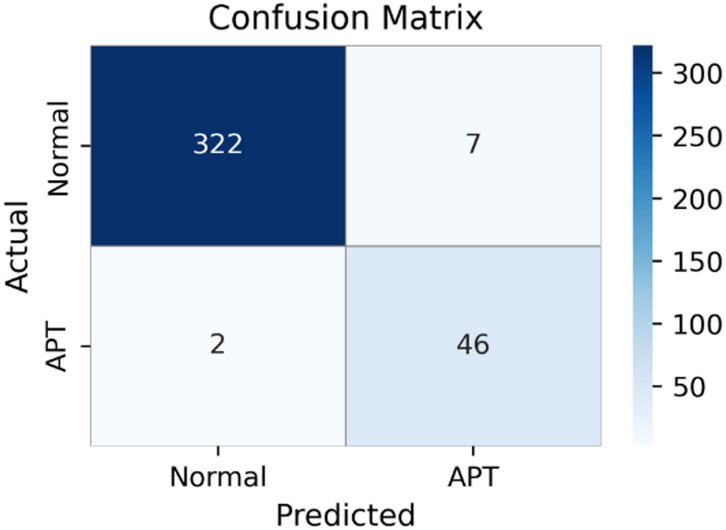
Confusion matrix of PCA-Transformer (K = 65), indicating higher misclassification than ET.

Figure 3 presents the confusion matrix of the PCA–Transformer model. The model correctly classifies 322 Normal samples and 46 APT samples, while producing 7 false positives and 2 false negatives. These results are consistent with the quantitative metrics reported in Table 3 and indicate relatively strong classification performance. Nevertheless, the presence of false negatives remains a concern in practical APT detection scenarios, as missed attacks may persist in the system. In addition, false positives can introduce unnecessary alerts, affecting operational efficiency.

Unlike compression-based approaches such as AE and PCA, ExtraTree emphasizes feature selection rather than purely dimensionality reduction. By leveraging decision-tree structures, ExtraTree identifies and removes irrelevant or noisy features while preserving those most informative for the classification task. This selective mechanism allows the downstream Transformer to focus on higher-quality inputs, contributing to improved overall performance.

From this experimental scenario, it can be concluded that ExtraTree plays a key role in the ET model by not only reducing dimensionality but also enhancing feature relevance, leading to more reliable and effective APT attack detection compared with alternative dimensionality reduction methods.

Second: Replacing Transformer with other embedding models.

In the third part of Scenario 1, we evaluate the effectiveness of replacing Transformer with other embedding models in the ET model. The ExtraTree component is fixed to ensure consistency in feature selection, while embedding models such as LSTM-GAN, BiLSTM-CGMS, and BiLSTM-Attention-CGMS are used to replace the Transformer. Detailed results are presented in Table 4.

**Table 4:** Comparison of classification performance for embedding models replacing transformer.

Embedding model	Accuracy	Precision	Recall	F1-Score
LSTM-GAN	0.9576	0.9606	0.9576	0.9586
BiLSTM-CGMS	0.9682	0.9707	0.9682	0.9650
BiLSTM-Attention-CGMS	0.9708	0.9727	0.9708	0.9714
Our	0.9933	0.9963	0.9890	0.9926

The results in Table 4 indicate that replacing the Transformer with LSTM-GAN leads to a noticeable reduction in performance, with Accuracy = 0.9576 and F1 = 0.9586. This may be attributed to the limited ability of LSTM-based models to capture complex and long-range dependencies beyond sequential patterns, which are common in network flow data.

When BiLSTM-CGMS is used, performance improves to Accuracy = 0.9682 and F1 = 0.9650, suggesting that modeling bidirectional dependencies provides more expressive representations than unidirectional LSTM. However, the absence of an explicit attention mechanism may limit the model’s ability to prioritize the most informative features, resulting in suboptimal performance compared with the Transformer-based design.

Among the alternative embedding approaches, BiLSTM-Attention-CGMS achieves the strongest results, with Accuracy = 0.9708 and F1 = 0.9714. This improvement suggests that incorporating an attention mechanism enables the model to better focus on relevant feature interactions while preserving sequential context. Nevertheless, its performance remains below that of the original ET model.

**Fig. 4:**
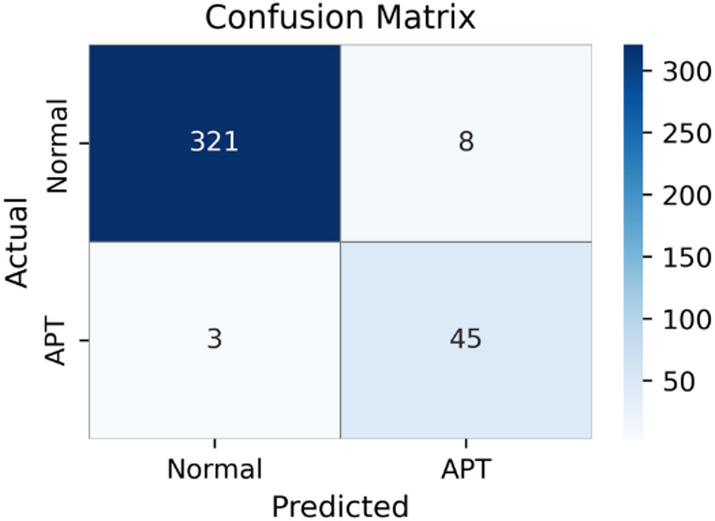
Confusion matrix of the BiLSTM-Attention-CGMS variant, suggesting competitive performance but lower accuracy than the Transformer-based configuration.

Figure 4 presents the confusion matrix for the BiLSTM-Attention-CGMS model. The model correctly classifies 321 Normal samples and 45 APT samples, while producing 8 false positives and 3 false negatives. These results are consistent with the quantitative metrics reported in Table 4, indicating reasonable detection capability. However, the presence of false negatives remains a concern in APT detection scenarios, as undetected attacks may persist in operational environments.

Although BiLSTM-Attention-CGMS achieves the best performance among the embedding alternatives, it still does not match the results obtained by the Transformer-based ET model. This observation suggests that the Transformer’s parallel processing capability and self-attention mechanism are particularly effective for modeling complex relationships in feature-selected flow data.

Overall, these findings indicate that while alternative embedding models can serve as viable replacements for Transformer to a certain extent, the original ET configuration remains the most effective in terms of classification performance and generalization. To further verify that the observed improvements are not dependent on a specific train–test split, we conduct an additional robustness analysis using 5-fold stratified cross-validation, as reported in Sect.  4.5.2.

#### End-to-end performance and robustness evaluation of ET-SDG

This subsection reports the end-to-end performance of the complete ET-SDG framework under a unified and statistically robust evaluation protocol. Unless otherwise stated, all subsequent experimental results in Sect.  4.5 are obtained using the same 5-fold cross-validation setting described here, with only point estimates reported for brevity.

To address the concern that the performance gains reported in Sect.  4.5.1 might be influenced by a favorable data partition, we further evaluate the robustness of the ET-SDG using a 5-fold stratified cross-validation protocol. Specifically, in each fold, the dataset is split into 80% for training and 20% for testing at the IP-pair level, while preserving the class distribution. This procedure is repeated five times with different data partitions, and all reported results correspond to the mean and standard deviation across folds.

Consistent with the pipeline design, feature aggregation and CGMS-based synthesis are applied only on the training set after data splitting, while the test set remains untouched and is reserved exclusively for evaluation.

Importantly, this analysis is designed to assess the stability of the ET-based feature extraction module within the end-to-end ET-SDG pipeline. To this end, the same classifier and experimental settings as in Sect.  4.5.1 are kept fixed across all folds, ensuring that observed performance variations primarily reflect differences in feature extraction rather than changes in downstream classification.

Tables 5, 6, and 7 summarize the updated 5-fold stratified cross-validation results (80/20 split per fold), including overall performance, per-class evaluation metrics, and class-specific ROC-AUC and PR-AUC values, with a particular emphasis on the minority MALICIOUS (APT) class. As shown, the ET-SDG framework consistently achieves strong performance across different folds, with low variance for all evaluation metrics. In particular, the overall F1-score exhibits a standard deviation of only 0.0057, while the PR-AUC standard deviation remains around **0.0013**, indicating stable and reliable discrimination capability under different 80/20 train–test splits.

**Table 5:** Overall performance of the ET-SDG under 5-fold stratified cross-validation (80/20 split per fold).

Metric	Mean ± Std	95% CI
Accuracy	0.9933 ± 0.0051	[0.9870, 0.9997]
Precision (overall)	0.9963 ± 0.0052	[0.9898, 1.0000]
Recall (overall)	0.9890 ± 0.0090	[0.9778, 1.0000]
F1-score (overall)	0.9926 ± 0.0057	[0.9855, 0.9997]
Specificity	0.9970 ± 0.0043	[0.9916, 1.0000]
G-Mean	0.9929 ± 0.0054	[0.9862, 0.9997]
MCC	0.9866 ± 0.0104	[0.9737, 0.9995]
ROC-AUC	0.9986 ± 0.0014	[0.9968, 1.0000]
PR-AUC	0.9987 ± 0.0013	[0.9971, 1.0000]

The consistently low standard deviation across folds demonstrates that the performance improvements achieved by the ET feature extraction module are not dependent on a particular 80/20 train–test split. These results suggest that the gains observed in Sect.  4.5.1 are statistically stable and are unlikely to be driven by a single favorable data partition.

**Table 6:** Per-class performance under 5-fold cross-validation.

Class	Precision (Mean ± Std)	95% CI	Recall (Mean ± Std)	95% CI	F1 (Mean ± Std)	95% CI
BENIGN	0.9910 ± 0.0074	[0.9818, 1.0000]	0.9970 ± 0.0043	[0.9916, 1.0000]	0.9939 ± 0.0047	[0.9881, 0.9997]
MALICIOUS (APT)	0.9963 ± 0.0052	[0.9898, 1.0000]	0.9890 ± 0.0090	[0.9778, 1.0000]	0.9926 ± 0.0057	[0.9855, 0.9997]

Table 6 further breaks down the results by class, which is critical for security detection where minority-class performance determines practical utility. For the MALICIOUS (APT) class, the model achieves high Precision = 0.9963 and strong Recall = 0.9890, resulting in an F1-score of 0.9926 with low standard deviation across folds. This implies that the proposed approach maintains both low false alarms (high precision) and high attack sensitivity (high recall) consistently under different splits. Meanwhile, the BENIGN class also achieves high Recall = 0.9970, reflecting reliable identification of normal traffic and supporting stable operational behavior.

**Table 7:** Per-class AUC (5-fold CV).

Class	ROC-AUC (Mean ± Std)	95% CI	PR-AUC (Mean ± Std)	95% CI
BENIGN	0.9986 ± 0.0014	[0.9968, 1.0000]	0.9984 ± 0.0017	[0.9963, 1.0000]
MALICIOUS (APT)	0.9986 ± 0.0014	[0.9968, 1.0000]	0.9987 ± 0.0013	[0.9971, 1.0000]

As shown in Table 7, both ROC-AUC and PR-AUC remain consistently close to 1.0 across classes, with very small variance. Notably, the MALICIOUS (APT) PR-AUC reaches 0.9987, which is particularly informative under class imbalance because it reflects precision–recall trade-offs for the positive (attack) class. Together, these AUC results corroborate that the model’s ranking and decision quality remain highly stable across folds, reinforcing the robustness conclusions drawn from Tables 5 and 6. Overall, these results demonstrate that the proposed ET-SDG framework maintains stable end-to-end performance under varying data partitions, supporting its robustness and practical applicability in real-world APT detection scenarios.

#### Evaluation of the ET-SDG model in APT IP classification

After a detailed analysis in Scenario 1 regarding the roles of components such as ExtraTree and Transformer, we continue with Scenario 2, focusing on evaluating the role of the CGMS-Attention model. The previous modules in the ET-SDG will be retained to address data imbalance and classify IP addresses.

##### ARQ2.1: comparison of CGMS-attention model with other methods

**Fig. 5:**
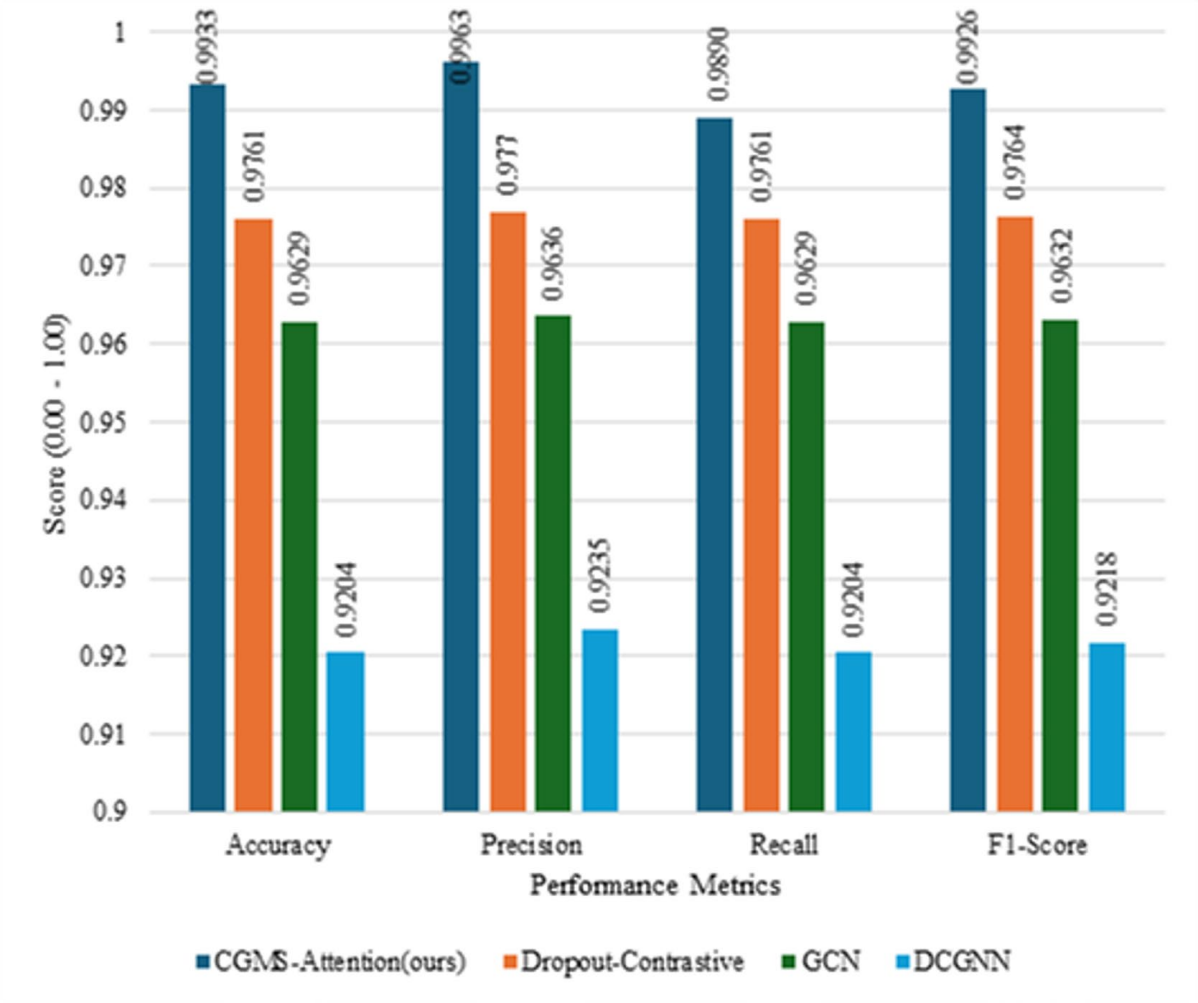
Performance comparison between the CGMS-Attention variant and alternative IP-level detection methods, showing consistent improvements across multiple metrics.

In this scenario, we compare the performance of the CGMS-Attention model with several other methods, including Dropout_Contrastive, GCN (Graph Convolutional Network), and DCGNN (Dynamic Convolutional Graph Neural Network). Detailed results are presented in Fig. 5.

As shown in Fig. 5, Dropout-Contrastive achieves the strongest performance among the alternative methods, with Accuracy = 0.9761 and F1 = 0.9764, which is relatively close to the performance of the CGMS-Attention-based model observed in previous experiments. This suggests that combining dropout regularization with contrastive learning can be effective for feature representation in network traffic data.

In contrast, the GCN model attains Accuracy = 0.9629 and F1 = 0.9632, which are lower than those achieved by CGMS-Attention. This performance gap may be attributed to GCN’s reliance on graph structural relationships, which may not sufficiently capture complex contextual and temporal characteristics inherent in network flow data.

The DCGNN model yields the lowest performance among the compared methods, with Accuracy = 0.9204 and F1 = 0.9218. Although DCGNN is designed to model dynamic graph structures, its limited ability to learn higher-order dependencies and generalizable representations appears to constrain its effectiveness in this task.

**Fig. 6:**
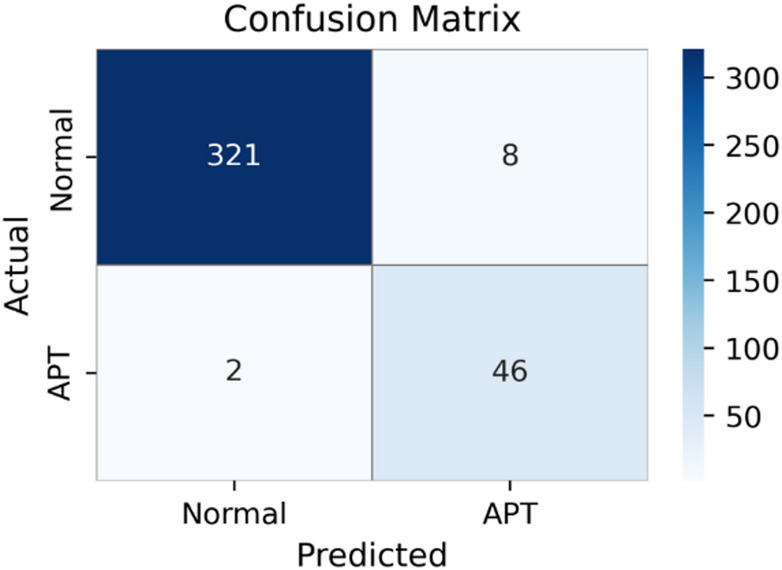
Confusion matrix of Dropout-Contrastive, illustrating residual false alarms and missed detections.

Figure 6 presents the confusion matrix for the Dropout-Contrastive model, which achieves the best performance among the alternative methods in Scenario 2. The model correctly classifies 321 Normal samples and 46 APT samples, while producing 8 false positives and 2 false negatives. These results are consistent with the quantitative metrics reported in Fig. 5 and indicate strong detection capability. However, the presence of false positives suggests that some false alerts may still occur in practical deployments, which could impact operational efficiency.

Overall, while Dropout-Contrastive demonstrates competitive performance and may serve as a viable alternative, both GCN and DCGNN fall noticeably behind the CGMS-Attention-based approach. These findings suggest that CGMS-Attention plays an important role in modeling rare and complex attack patterns, thereby contributing to improved IP-level APT detection within the ET-SDG framework.

##### ARQ2.2: evaluating the role of each component in CGMS-attention

**Fig. 7:**
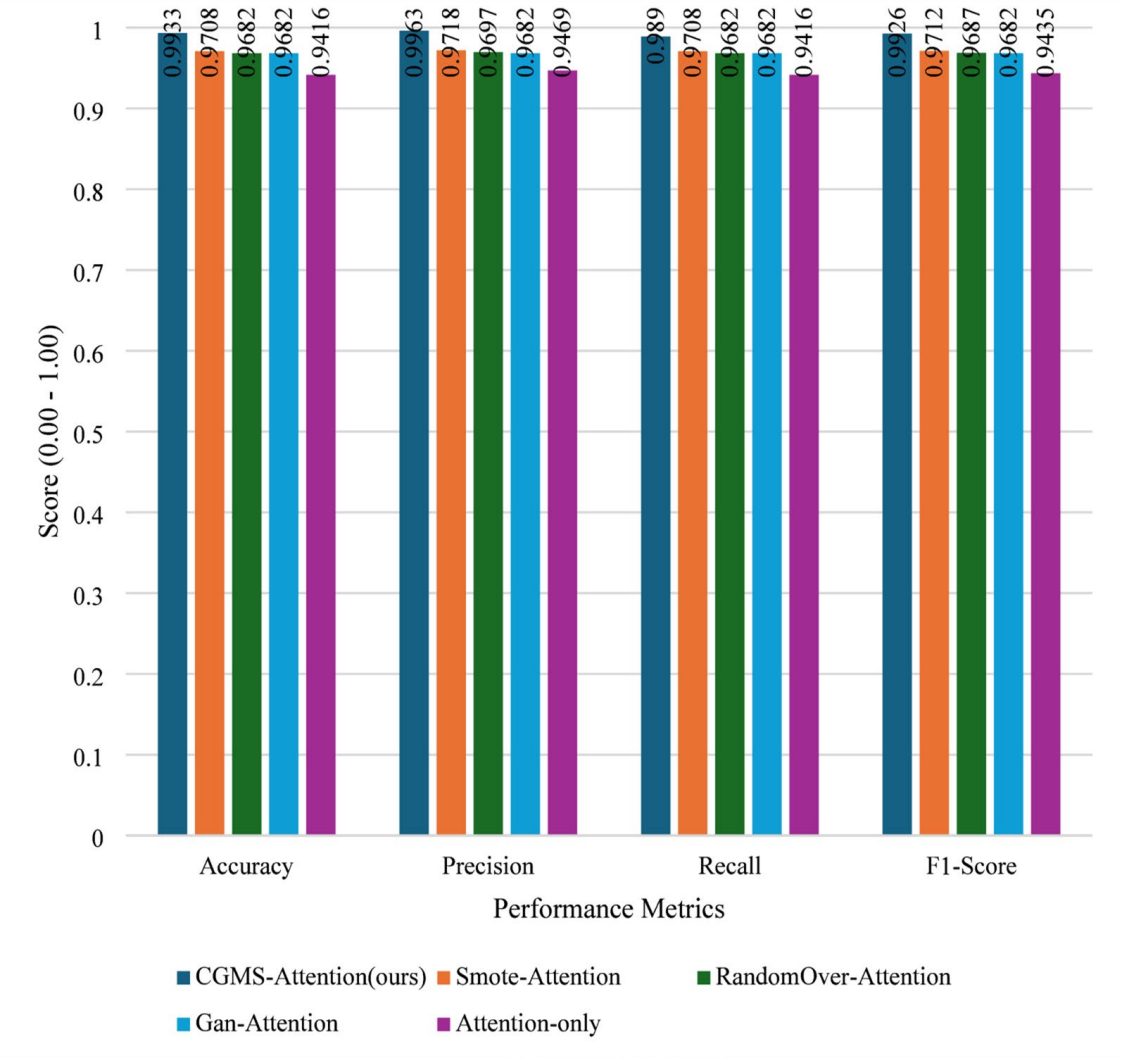
Ablation study on augmentation and attention, where CGMS-Attention achieves the strongest overall performance and SMOTE-Attention yields more missed attacks.

To evaluate the important role of CGMS-Attention in detecting APT attacks, we conducted a series of experiments comparing the performance of the original model with variations that either did not use CGMS or replaced CGMS with other data generation methods. The experimental results, presented in Fig. 7, show a clear difference in accuracy between the methods.

As shown in Fig. 7, removing both CGMS and the Attention mechanism (Attention-only) leads to a clear degradation in performance, with Accuracy = 0.9416 and Recall = 0.9416. This reduction suggests that, under severe class imbalance, the absence of both conditional data generation and attention-based feature weighting limits the model’s ability to accurately identify APT attacks.

When CGMS is replaced with a conventional GAN (GAN-Attention), performance improves to Accuracy = 0.9682. However, the corresponding Precision, Recall, and F1-score remain at 0.9682, which is lower than the configuration using CGMS-Attention. This observation suggests that although GAN-based augmentation can alleviate data imbalance to some extent, the lack of explicit label conditioning during generation may restrict the usefulness of the synthesized samples for downstream classification.

We further evaluate SMOTE-Attention, in which CGMS is replaced by SMOTE for minority data augmentation. This variant achieves Accuracy = 0.9708, with Precision = 0.9718, Recall = 0.9708, and F1 = 0.9712, representing the strongest performance among the alternative methods. These results indicate that SMOTE can generate informative synthetic samples and effectively improve detection capability. Nevertheless, SMOTE does not explicitly model the conditional distribution of minority classes. In contrast, CGMS, as a cGAN-based approach, aims to generate label-consistent samples that better preserve class-specific characteristics under severe imbalance.

Another replacement strategy, RandomOver-Attention, shows moderate improvement over the Attention only model, reaching Accuracy = 0.9682. However, its Precision, Recall, and F1-score remain lower than those of SMOTE-Attention. This suggests that simple random oversampling, which duplicates existing minority samples, may be less effective than synthetic data generation methods that introduce additional variability and structure.

**Fig. 8:**
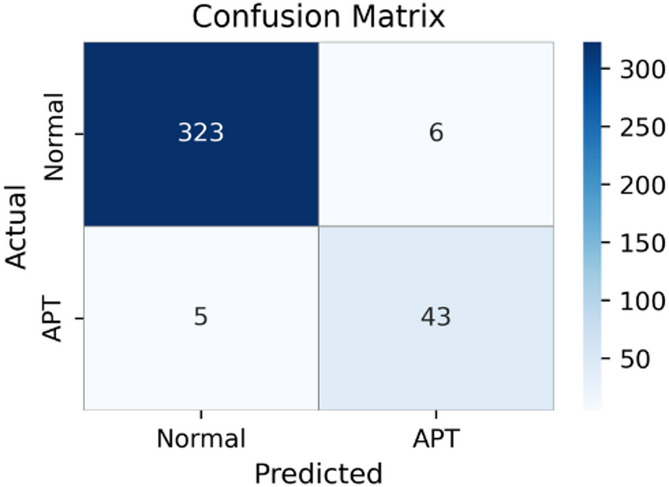
Confusion matrix of the SMOTE-Attention variant, showing improved detection compared to removing augmentation while still missing more APT samples than CGMS-Attention.

Figure 8 presents the confusion matrix for the SMOTE-Attention model. The model correctly classifies 323 Normal samples and 43 APT samples, while producing 6 false positives and 5 false negatives. These results demonstrate that SMOTE-Attention effectively balances the data and improves overall detection performance. However, the relatively higher number of false negatives compared with CGMS-Attention suggests that SMOTE-generated samples may not fully capture the most discriminative characteristics of APT attacks.

Overall, these results indicate that CGMS-Attention plays an important role in balancing data and enhancing detection performance. While SMOTE-Attention represents the strongest alternative among the evaluated replacements, it does not fully match the effectiveness of CGMS-Attention in generating representative minority samples. Meanwhile, GAN-Attention and RandomOver-Attention improve performance compared to removing data generation entirely, but their gains remain limited. Collectively, these findings suggest that combining CGMS with an Attention mechanism provides a more effective solution for learning from highly imbalanced data in APT attack detection tasks.

#### Comparison with other studies and approaches

In this scenario, we compare modern network attack detection methods to evaluate the performance of the ET-SDG in detecting fast attacks and APT attacks. These models were selected for their availability and effectiveness in detecting network attacks across various types of data, including imbalanced and complex data. Table 8 below lists the detailed results for this scenario.

**Table 8:** Comparative analysis of detection methods.

Model	Accuracy	Precision	Recall	F1-Score
CNN-LSTM-Attention^7^	0.9310	0.9310	0.9310	0.9310
Cnn-Lstm^35^	0.9443	0.9438	0.9443	0.9440
CNN-BiLSTM-Attention^36^	0.9735	0.9735	0.9735	0.9735
ALLKNN-LightGBM^37^	0.9732	0.9731	0.9742	0.9740
Bilstm-Attention-Dropout^8^	0.9761	0.9761	0.9761	0.9753
ACG‑BT^24^	0.9945	0.9811	0.9858	0.9835
MCG^23^	0.9732	0.9731	0.9742	0.9740
Our	0.9933	0.9963	0.9890	0.9926

The results from the table show a clear difference in classification performance among the compared models, particularly when evaluating metrics such as accuracy, precision, recall, and F1-score. Each method has its strengths and limitations, reflecting varying degrees of suitability when applied to the network attack detection problem. The CNN-LSTM-Attention model^7^ shows the lowest performance, with accuracy only reaching 0.9310. This can be explained by the fact that CNN only helps the model learn local spatial features and does not capture long-term sequential dependencies in the data. While LSTM can improve this to some extent by learning sequential dependencies, the combination is not strong enough to cope with complex attacks in the network environment, especially when handling imbalanced data. The Attention mechanism in this case also does not optimize the selection of important information.

The CNN-LSTM model^35^ improves slightly over the version with Attention, achieving an accuracy of 0.9443. However, the difference between these two models is not large, as both still struggle with learning long-term sequential patterns, and CNN only processes local spatial features.

Next, the model^36^ achieves significantly higher accuracy, with an accuracy of 0.9735. The use of BiLSTM helps the model learn sequential patterns from both the forward and backward directions, and the combination with Attention improves the focus on the most important features. However, there are still limitations due to CNN only learning spatial features at the local level.

The method in^37^ achieved an accuracy of 0.9732, with an F1-score of 0.9740, showing good performance in detecting fast attacks. This method primarily optimizes for handling imbalanced data, thanks to the combination of ALLKNN and LightGBM. However, the lack of sequential and spatial feature learning reduces the ability to detect complex attacks, leading to lower results compared to Attention-based models.

Finally, the BiLSTM-Attention-Dropout model^8^ achieved the highest accuracy (0.9761). The use of BiLSTM allows the model to learn both forward and backward sequential relationships, while Attention helps the model focus on the most important features. The Dropout mechanism helps reduce overfitting, enabling the model to maintain better performance. However, the difference between precision (0.9641) and recall (0.9750) indicates that the model is still not fully balanced, particularly when handling complex and imbalanced attacks.

Our ET-SDG model has addressed these weaknesses by learning sequential features well with Transformer and using a conditional data generation mechanism to tackle the issue of imbalanced data. As a result, ET-SDG achieves competitive performance and shows stable results under the evaluated experimental settings, suggesting that it is a suitable candidate for network attack detection. These observations are consistent with the results reported in Sect.  4.3, further confirming the limitations of CNN- and LSTM-based models when handling complex and imbalanced sequential data. In particular, CNN-LSTM and CNN-LSTM-Attention architectures struggle to fully capture long-range temporal dependencies and complex feature interactions, leading to lower classification accuracy. Although ALLKNN-LightGBM shows improved performance under class imbalance, it still exhibits limitations in balancing precision and recall for more complex attack patterns.

### Discussion

The experimental results from the three scenarios presented above indicate that ET-SDG performs competitively on the evaluated benchmarks. The results further suggest that the selected components make complementary contributions to the overall pipeline, as supported by the ablation and comparative analyses. In this discussion, we provide additional analysis to explain why ET-SDG may help mitigate two persistent challenges in prior APT detection pipelines, namely feature representation learning from complex flows and learning under severe class imbalance. Specifically:

#### The validity of choosing transformer and CGMS for the ET-SDG model

It can be observed that in APT detection systems, Transformer has proven effective in feature extraction due to its self-attention mechanism, allowing the model to learn the relationships between packets in network flows. At the same time, CGMS plays an important role in generating synthetic data to address the imbalance between normal network traffic and attack traffic. However, to accurately assess the contribution of Transformer and CGMS to the model’s classification ability, we conducted a scenario where both components were removed. Table 9 presents the results of the model when Transformer was not used for feature extraction and CGMS was not applied to generate attack data.

**Table 9:** Evaluation results of the model variant without transformer and CGMS.

Model	Accuracy	Precision	Recall	F1-score
ET-SDG (without Transformer and CGMS)	0.9443	0.9423	0.9443	0.9430

As shown in Table 9, removing both the Transformer and CGMS components leads to a noticeable degradation in performance. The model achieves an accuracy of 0.9443, a precision of 0.9423, a recall of 0.9443, and an F1-score of 0.9430. These results are substantially lower than those of the full ET-SDG model, highlighting the critical contribution of both components to the overall framework.

**Fig. 9:**
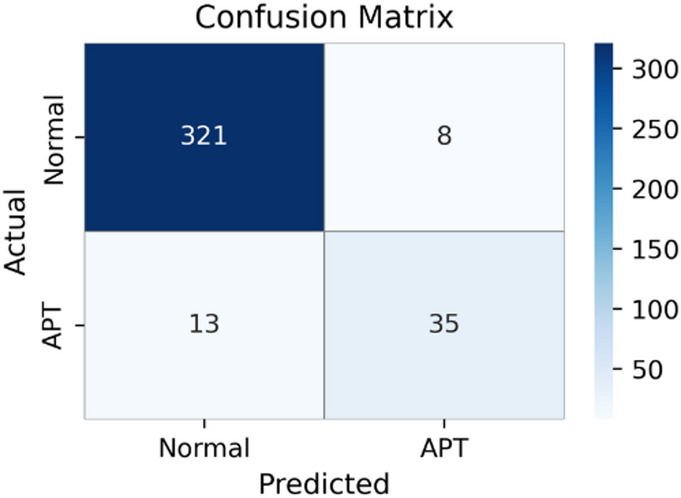
Confusion matrix of the model variant without Transformer and CGMS, showing an increased number of false negatives when both components are removed.

Figure 9 provides a visual illustration of this degradation. The model correctly classifies 321 Normal samples and 35 APT samples, while producing 8 false positives and 13 false negatives. The relatively high number of false negatives highlights a key limitation of this configuration, as missed APT attacks can remain undetected in real-world deployments.

This behavior suggests that, without the Transformer module, the model has limited capability to capture complex contextual relationships within network flow data, leading to weaker discrimination between benign and malicious patterns. At the same time, the absence of CGMS reduces the model’s ability to effectively learn from imbalanced data, resulting in a bias toward the majority class. Together, these factors help explain both the elevated false-negative rate and the persistence of false positives observed in Fig. 9. Overall, this ablation analysis provides additional evidence that Transformer and CGMS play complementary roles within ET-SDG: Transformer enhances contextual feature modeling, while CGMS improves learning under class imbalance. Their combined use contributes to more reliable and robust APT attack detection.

#### Effectiveness in the selection and optimization of parameters in the ET-SDG model

In this paper, we have fine-tuned all the parameters of each component in the ET-SDG model. Below are the results that show how the experimental results change when these parameters are adjusted.

First: Parameter Selection and Configuration for ExtraTree Algorithm.

The experimental results in Table 10 describe how APT IP detection changes when the parameters of the ExtraTree algorithm are varied. To determine an appropriate feature count, we first performed a coarse-grained search from 10 to 76 with a step size of 5. This preliminary exploration indicated that performance remains relatively strong within the 60–70 range, with a noticeable peak around 65 features. We then refined the search by using a step size of 1 within [60, 70] to identify a suitable configuration.

**Table 10:** APT IP detection results with different ExtraTree parameters.

num_features	Accuracy (mean ± std)	Precision (mean ± std)	Recall (mean ± std)	F1 (mean ± std)
60	0.9910 ± 0.0038	0.9927 ± 0.0058	0.9875 ± 0.0092	0.9900 ± 0.0043
61	0.9913 ± 0.0059	0.9934 ± 0.0080	0.9875 ± 0.0103	0.9904 ± 0.0066
62	0.9920 ± 0.0043	0.9948 ± 0.0056	0.9875 ± 0.0085	0.9911 ± 0.0048
63	0.9907 ± 0.0045	0.9934 ± 0.0066	0.9860 ± 0.0099	0.9897 ± 0.0050
64	0.9917 ± 0.0043	0.9941 ± 0.0056	0.9875 ± 0.0103	0.9908 ± 0.0047
65	0.9933 ± 0.0051	0.9963 ± 0.0052	0.9890 ± 0.0090	0.9926 ± 0.0057
66	0.9920 ± 0.0048	0.9956 ± 0.0061	0.9868 ± 0.0089	0.9911 ± 0.0053
67	0.9917 ± 0.0046	0.9956 ± 0.0048	0.9860 ± 0.0095	0.9908 ± 0.0051
68	0.9920 ± 0.0046	0.9956 ± 0.0048	0.9868 ± 0.0092	0.9911 ± 0.0052
69	0.9913 ± 0.0038	0.9941 ± 0.0049	0.9868 ± 0.0076	0.9904 ± 0.0042
70	0.9910 ± 0.0038	0.9941 ± 0.0049	0.9860 ± 0.0084	0.9900 ± 0.0043

Significant values are in bold.

As shown in Table 10, varying the number of selected features influences performance across Accuracy, Precision, Recall, and F1-score. Within the explored range, the configuration with 65 features yields the strongest overall results, achieving Accuracy = 0.9933, Precision = 0.9963, Recall = 0.9890, and F1 = 0.9926. Neighboring settings exhibit slightly lower F1-scores (e.g., 0.9900 at 60 and 0.9900 at 70), suggesting that selecting 65 features provides a reasonable trade-off between retaining informative attributes and limiting feature redundancy.

Second: Parameter Selection and Configuration for Transformer.

In this experimental scenario, we evaluated the impact of the number of Multi-Head Attention units in the Transformer architecture on the model’s classification ability.

**Table 11:** Results of the model with different transformer parameters.

Num_Multihead	Accuracy (mean ± std)	Precision (mean ± std)	Recall (mean ± std)	F1 (mean ± std)
4	0.9893 ± 0.0051	0.9948 ± 0.0033	0.9816 ± 0.0107	0.9881 ± 0.0057
6	0.9893 ± 0.0056	0.9926 ± 0.0037	0.9838 ± 0.0109	0.9882 ± 0.0063
8	0.9933 ± 0.0051	0.9963 ± 0.0052	0.9890 ± 0.0090	0.9926 ± 0.0057
10	0.9897 ± 0.0055	0.9963 ± 0.0027	0.9809 ± 0.0099	0.9885 ± 0.0061
12	0.9880 ± 0.0040	0.9933 ± 0.0048	0.9802 ± 0.0085	0.9867 ± 0.0045
16	0.9903 ± 0.0055	0.9970 ± 0.0032	0.9816 ± 0.0094	0.9892 ± 0.0061

Significant values are in bold.

The results in Table 11 indicate that when the number of Multi-Head Attention units is 8, the model achieves the strongest performance, with Accuracy = 0.9933, Precision = 0.9963, Recall = 0.9890, and F1 = 0.9926. This behavior suggests that using 8 attention heads provides sufficient capacity to model complex dependencies in network data without introducing unnecessary redundancy.

When the number of attention heads is reduced to 4, performance decreases, with F1 = 0.9881, indicating that limited attention capacity may constrain the model’s ability to capture diverse feature interactions. Increasing the number of heads to 6 leads to a slight improvement F1 = 0.9882, but the performance remains below that achieved with 8 heads. Conversely, further increasing the number of attention heads beyond 8 does not yield additional gains. In particular, configurations with 10, 12, and 16 heads result in F1-scores of 0.9885, 0.9867, and 0.9892, respectively. This trend suggests that increasing model complexity beyond a certain point may offer diminishing returns and does not necessarily improve generalization in this setting.

Third: Parameter Selection and Configuration for CGMS.

Among the hyperparameters of CGMS, the latent dimension size plays a central role in controlling the capacity of the conditional data generation process. In this experiment, we evaluate how varying the latent dimension affects the model’s ability to generate informative minority-class samples and, consequently, its classification performance. The corresponding results are reported in Table 12.

**Table 12:** Experimental results of the model with different CGMS parameters.

Num_Latent-Dim	Accuracy (mean ± std)	Precision (mean ± std)	Recall (mean ± std)	F1 (mean ± std)
50	0.9887 ± 0.0051	0.9940 ± 0.0042	0.9809 ± 0.0099	0.9874 ± 0.0057
100	0.9933 ± 0.0051	0.9963 ± 0.0052	0.9890 ± 0.0090	0.9926 ± 0.0057
150	0.9897 ± 0.0063	0.9955 ± 0.0049	0.9816 ± 0.0107	0.9885 ± 0.0070
200	0.9897 ± 0.0057	0.9941 ± 0.0042	0.9831 ± 0.0118	0.9885 ± 0.0064

Significant values are in bold.

As shown in Table 8, setting the latent dimension to 100 yields the strongest overall performance, with Accuracy = 0.9933, Precision = 0.9963, Recall = 0.9890, and F1 = 0.9926. This result suggests that a latent space of this size provides sufficient representational capacity for CGMS to capture meaningful variations within the minority class while maintaining stable training behavior.

When the latent dimension is reduced to 50, performance decreases, with F1 = 0.9874, indicating that an overly compact latent space may limit the generator’s ability to encode diverse attack patterns. Conversely, increasing the latent dimension to 150 or 200 does not lead to further improvements, with both settings achieving F1 = 0.9885. This trend suggests that excessively large latent spaces may introduce redundant degrees of freedom, which do not translate into more discriminative synthetic samples and may reduce the effectiveness of conditional generation.

Overall, these results indicate that a latent dimension of 100 provides a balanced configuration, offering an effective trade-off between representational capacity and generalization for CGMS within the ET-SDG framework.

**Fig. 10:**
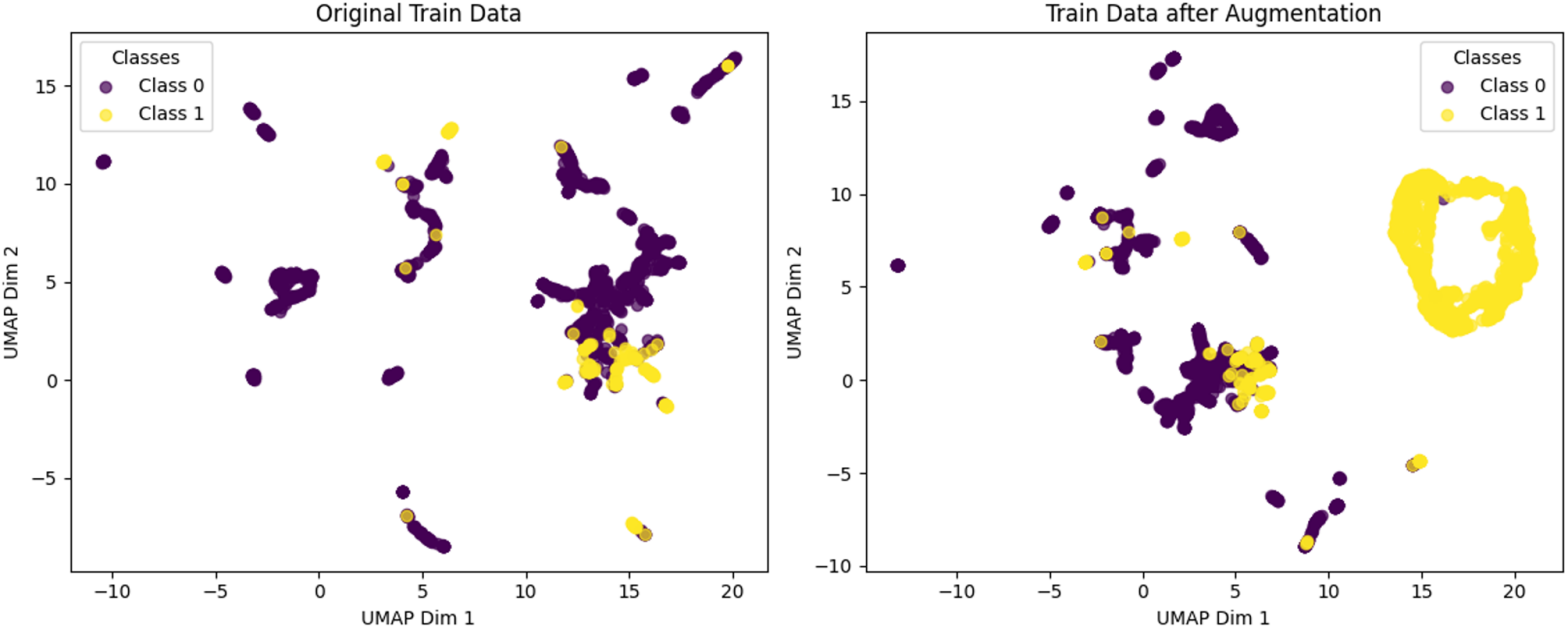
Data before and after augmentation.

To qualitatively illustrate the effect of conditional data generation, we further visualize the data distribution using UMAP (Uniform Manifold Approximation and Projection), as shown in Fig. 10.

Figure 10 compares the training data distribution before (left) and after (right) applying CGMS-based conditional data augmentation. In the original data, the minority APT class is sparsely represented relative to the normal class, reflecting a pronounced class imbalance. After augmentation, additional APT samples are introduced, leading to a denser and more evenly distributed representation in the feature space. Notably, the synthetic samples extend the coverage of the minority class rather than merely duplicating existing points, suggesting that CGMS captures meaningful variations within the attack class.

This qualitative observation is consistent with the quantitative improvements reported in Table 8 and indicates that CGMS helps alleviate data imbalance while preserving diversity in minority-class representations. Such behavior is particularly important for APT attack detection, where rare but structurally diverse attack patterns must be learned effectively. Together, these results further support the role of CGMS in improving model robustness and generalization under imbalanced data conditions.

Fourth: Changing the Size of Query, Key, and Value Vectors in Attention.

In this experiment, we evaluate the impact of the dimensionality of the Query, Key, and Value (Q–K–V) vectors in the Attention mechanism on overall model performance. The corresponding results are summarized in Table 13.

**Table 13:** Evaluation results of the model with changing attention values.

num_nodes	Accuracy (mean ± std)	Precision (mean ± std)	Recall (mean ± std)	F1 (mean ± std)
20-20-20	0.9913 ± 0.0058	0.9934 ± 0.0080	0.9875 ± 0.0089	0.9904 ± 0.0064
40-40-40	0.9933 ± 0.0051	0.9963 ± 0.0052	0.9890 ± 0.0090	0.9926 ± 0.0057
60-60-60	0.9930 ± 0.0058	0.9956 ± 0.0066	0.9890 ± 0.0090	0.9922 ± 0.0065
80-80-80	0.9917 ± 0.0069	0.9934 ± 0.0092	0.9882 ± 0.0088	0.9908 ± 0.0076
100-100-100	0.9927 ± 0.0047	0.9948 ± 0.0056	0.9890 ± 0.0090	0.9919 ± 0.0052

Significant values are in bold.

As shown in Table 13, the configuration with 40–40–40 achieves the strongest overall performance, with Accuracy = 0.9933, Precision = 0.9963, Recall = 0.9890, and F1 = 0.9926. This observation suggests that this vector size provides sufficient representational capacity for the Attention mechanism to model feature interactions in network traffic while maintaining a reasonable level of model complexity.

When the vector size is reduced to 20–20–20, performance decreases, with F1 = 0.9904, indicating that overly compact Q–K–V representations may limit the model’s ability to capture richer relationships among features. Increasing the vector size to 60–60–60 results in a slight reduction in performance F1 = 0.9922, while further increases to 80–80–80 and 100–100–100 yield F1-scores of 0.9908 and 0.9919, respectively. These results suggest that expanding the vector dimensionality beyond a certain point does not translate into consistent performance gains and may introduce unnecessary complexity.

Overall, these findings indicate that a Q–K–V dimensionality of 40–40–40 represents a balanced configuration in this setting, offering an effective trade-off between representational expressiveness and computational efficiency. This configuration therefore supports stable and accurate APT detection within the ET-SDG framework.

#### Evaluation on an additional large-scale imbalanced dataset

To further assess the generalization capability of the proposed approach, we conducted additional experiments on the NF-CSE-CIC-IDS2018-v2 dataset, which is significantly larger and exhibits a much higher degree of class imbalance. The characteristics of this dataset have been detailed and analyzed in^1^. The comparative results are summarized in Table 14.

**Table 14:** Performance comparison on the NF-CSE-CIC-IDS2018-v2 dataset.

Method	Accuracy	Precision	Recall	F1-score
E-GraphSAGE^40^	0.9320	0.9249	0.7340	0.7936
Anomal-E^41^	0.9157	0.8303	0.7222	0.7617
E-ResGAT^42^	0.9845	0.9734	0.9530	0.9629
FeCoGraph^43^	**0.9963**	0.9938	**0.9885**	**0.9911**
ACDF-mLSTM^1^	0.9946	0.9965	0.9778	0.9869
Our	0.9955	**0.9998**	0.9619	0.9804

Significant values are in bold.

As shown in Table 10, the ET-SDG achieves Accuracy = 0.9955 and Precision = 0.9998, indicating a very low false-alarm rate on this highly imbalanced dataset. Compared with graph-based methods such as E-GraphSAGE and Anomal-E, our approach demonstrates substantially stronger overall performance across all evaluation metrics.

When compared with more recent and competitive methods, including E-ResGAT, FeCoGraph, and ACDF-mLSTM, ET-SDG does not consistently achieve the highest F1-score. In particular, FeCoGraph attains a higher F1 = 0.9911, mainly due to its stronger recall (0.9885) on the minority class. In contrast, ET-SDG exhibits a more conservative detection behavior, characterized by very high precision (0.9998) and a comparatively lower recall (0.9619).

This trade-off suggests that, on extremely imbalanced large-scale datasets, the proposed approach prioritizes minimizing false positives, which can be desirable in practical deployment scenarios where excessive false alarms may overwhelm security analysts. At the same time, the recall achieved by ET-SDG remains competitive and substantially higher than that of several baseline methods.

Overall, the additional evaluation on NF-CSE-CIC-IDS2018-v2 provides further evidence that ET-SDG can maintain strong performance under a substantially larger and more severely imbalanced setting. Nevertheless, several practical limitations remain and are discussed in the next subsection.

#### Limitations

Despite the encouraging results, several limitations remain. First, the proposed ET-SDG framework introduces higher computational overhead than simple resampling-based pipelines with a single classifier, as it requires training both the conditional generation module and the sequential feature learning component. This may restrict applicability in highly resource-constrained environments or latency-critical deployments, where lightweight models are preferred.

Second, although we evaluated the framework on multiple public benchmarks, all experiments were conducted in an offline setting. The impact of concept drift, evolving attack behaviors, and long-term deployment dynamics has not been systematically investigated, and future work will consider online/streaming evaluation protocols and practical updating strategies (e.g., periodic retraining or continual adaptation).

Third, the current design focuses on flow-level features, which are widely available and compatible with encrypted traffic monitoring. Nevertheless, integrating complementary information sources—such as payload-derived signals when permissible, side-channel metadata, or self-supervised pretraining on large-scale unlabeled traffic—may further improve robustness to unseen or emerging attacks.

## Conclusion and future directions

With the goal of improving APT attack detection under complex traffic patterns and severe class imbalance, we developed ET-SDG as an integrated pipeline based on ensemble-learning-oriented design. The experimental results on the evaluated benchmarks suggest that ET-SDG can help mitigate two persistent challenges in APT detection: (i) learning informative representations from flow-network data and (ii) learning reliably under highly imbalanced class distributions. Specifically, for feature extraction and representation, ET-SDG combines ExtraTrees-based feature ranking with a Transformer-based encoder to capture contextual relationships among selected flow features. This design can reduce the impact of less-informative and potentially redundant features while preserving discriminative patterns relevant to APT behaviors, thereby supporting more effective detection under the evaluated settings. For class imbalance, the integration of CGMS-based (cGAN) augmentation with an attention-based classification/aggregation component provides a practical way to enrich minority-class training samples and to emphasize informative patterns in learned representations. The experimental results indicate that ET-SDG achieves competitive performance relative to the considered hybrid baselines across multiple evaluation metrics, particularly in imbalanced scenarios.

In future work, we plan to further strengthen the robustness and applicability of APT detection in real-world deployments. First, we will explore richer dependency modeling among flows (e.g., attention-based correlation modeling and graph-structured inference) to better capture cross-flow relationships. Second, we will investigate more robust and transferable representation learning strategies, including domain-adapted Transformer encoders and foundation-model-inspired embeddings tailored to network traffic. Finally, to better handle evolving attacker behaviors and data imbalance, we will consider continual learning and drift-aware evaluation, as well as advanced training strategies such as contrastive learning and knowledge distillation to improve generalization and stability.

## Data Availability

The datasets generated and (or) analysed during the current study are available from the corresponding author on reasonable request.
